# SLC3A2 N-glycosylation and Golgi remodeling regulate SLC7A amino acid exchangers and stress mitigation

**DOI:** 10.1016/j.jbc.2023.105416

**Published:** 2023-11-02

**Authors:** Cunjie Zhang, Massiullah Shafaq-Zadah, Judy Pawling, Geoffrey G. Hesketh, Estelle Dransart, Karina Pacholczyk, Joseph Longo, Anne-Claude Gingras, Linda Z. Penn, Ludger Johannes, James W. Dennis

**Affiliations:** 1 Lunenfeld-Tanenbaum Research Institute, Mount Sinai Hospital, Toronto Ontario, Canada; 2 Cellular and Chemical Biology Unit, Institut Curie, INSERM U1143, CNRS UMR3666, PSL Research University, Paris, France; 3 Princess Margaret Cancer Centre, University Health Network, Toronto, Ontario, Canada; 4 Department of Medical Biophysics, University of Toronto, Toronto, Ontario, Canada; 5 Department of Molecular Genetics, University of Toronto, Toronto, Ontario, Canada; 6 Department of Laboratory Medicine and Pathobiology, University of Toronto, Toronto, Ontario, Canada

**Keywords:** amino acids, transporters N-glycosylation, galectins, metabolism, evolution

## Abstract

Proteostasis requires oxidative metabolism (ATP) and mitigation of the associated damage by glutathione, in an increasingly dysfunctional relationship with aging. SLC3A2 (4F2hc, CD98) plays a role as a disulfide-linked adaptor to the SLC7A5 and SLC7A11 exchangers which import essential amino acids and cystine while exporting Gln and Glu, respectively. The positions of N-glycosylation sites on SLC3A2 have evolved with the emergence of primates, presumably in synchrony with metabolism. Herein, we report that each of the four sites in SLC3A2 has distinct profiles of Golgi-modified N-glycans. N-glycans at the primate-derived site N381 stabilized SLC3A2 in the galectin-3 lattice against coated-pit endocytosis, while N365, the site nearest the membrane promoted glycolipid-galectin-3 (GL-Lect)-driven endocytosis. Our results indicate that surface retention and endocytosis are precisely balanced by the number, position, and remodeling of N-glycans on SLC3A2. Furthermore, proteomics and functional assays revealed an N-glycan-dependent clustering of the SLC3A2∗SLC7A5 heterodimer with amino-acid/Na^+^ symporters (SLC1A4, SLC1A5) that balances branched-chain amino acids and Gln levels, at the expense of ATP to maintain the Na^+^/K^+^ gradient. In replete conditions, SLC3A2 interactions require Golgi-modified N-glycans at N365D and N381D, whereas reducing N-glycosylation in the endoplasmic reticulum by fluvastatin treatment promoted the recruitment of CD44 and transporters needed to mitigate stress. Thus, SLC3A2 N-glycosylation and Golgi remodeling of the N-glycans have distinct roles in amino acids import for growth, maintenance, and metabolic stresses.

The uptake of carbohydrates, amino acids (AA), and lipids by membrane transporters fuels ATP production by oxidative phosphorylation, growth, heat, and damaging entropy in the form of reactive oxygen species (ROS). Control of these forces requires posttranslational modifications of signaling intermediates in the regulation of gene expression and metabolism (1, 2). This includes N-glycosylation in the endoplasmic reticulum (ER), and Golgi remodeling of the N-glycan on proteins of the secretory pathway (3). Both steps require uridine diphosphate N-acetylglucosamine (UDP-GlcNAc) generated by the hexosamine biosynthetic pathway (HBP) from glucose, glutamine (Gln), and acetyl-coenzyme A, thus competing for these substrates with glycolysis, glutaminolysis, and sterol turnover (4, 5). Reduced flux of these metabolites to the N-glycosylation of newly synthesized proteins in the ER activates the unfolded protein response (6, 7, 8). In replete conditions, glycoproteins transit from the ER to Golgi, where N-glycans are trimmed and remodeled with sequences that bind galectins, a family of β-galactoside binding proteins. Branching N-acetylglucosaminyltransferases (MGAT1, 2, 4 and 5) add GlcNAc followed by substitution with galactose generating up to four Galβ1-4GlcNAc1- branches on the trimannosyl core (9). Branched N-glycans are found widely on receptor kinases (10, 11, 12), integrins (13), glucagon receptor (14), and nutrient transporters (15, 16, 17). The more branched N-glycans are higher affinity ligands for the carbohydrate-binding domain of galectin-3 (Gal-3), the only member of the family that also has an intrinsically disordered self-associating C-terminal tail (18, 19, 20). Gal3 cross-linking of membrane glycoproteins induces phase transition at critical concentrations (termed galectin lattice) which slows the diffusion of receptors and nutrient transporters into clathrin-coated pits, caveolae, and macropinosomes, thereby slowing loss to endocytosis (10, 12, 21, 22). Gal3 also drives glycolipid-dependent formation of tubular endocytic pits according to the GlycoLipid-Lectin (GL-Lect) hypothesis, from which clathrin-independent endocytic carriers are generated for the uptake of glycoproteins and their subsequent endosomal sorting (23, 24, 25).

A genome-wide CRISPR screen in mammalian cells has revealed correlated essentiality between fatty acid synthase (FASN), the N-glycosylation pathway, and exogenous lipid uptake by the heavily N- glycosylated LDL receptor (26). *In vivo*, Mgat5 deficient mice are resistant to weight gain on a high-fat diet with improved insulin sensitivity (27), whereas transgenic mice overexpressing Mgat5 in the liver, display obesity, steatosis, and insulin resistance (28, 29). Dietary supplementation with GlcNAc also increases weight gain in adult wild type, but not in Mgat5^−/−^ mice where a small increase in fat mass was offset by a decline in lean tissue mass (30). Indeed, Mgat5^−/−^ mice on a normal diet age prematurely show steatosis and muscle weakness (27). Taken together, these studies suggest that nutrient uptake may be dependent on nutrient flux to HBP and N-glycan branching on nutrient transporters. Indeed, GlcNAc salvage into hepatic UDP-GlcNAc increased N-glycan branching, and Gln levels relative to essential amino acids (EAA) (30). Gln is a limiting substrate of the *de novo* HBP and N-glycan branching (16, 31), generating positive feedback to transporters that drive EAA uptake and lipogenesis (17, 32).

The evolution of N-glycosylation sites on SLC3A adaptors and SLC7A transporters suggests a critical but poorly understood role for N-glycans. SLC3A2 (4F2hc, CD98) is a type 2 transmembrane glycoprotein that forms disulfide-linked heterodimers (indicated by ∗) with the AA exchangers, SLC7A5, -6, -7, -8, -9, -10, -11) (33, 34), bringing N-glycans to the heterodimer, as the exchangers are not N-glycosylated (Fig. 1, *A* and *B*). In contrast, SLC7A1,-2,-3,-4 paralogues are N-glycosylated at conserved positions and do not heterodimerize with an adaptor. SLC7A5, -7, -8, -10 balance intracellular EAAs with the demands of metabolism. However, intracellular Gln also acts as an export substrate that drives EAA levels higher, and in turn Rag GTPase/mTOR signaling (32, 35). Similarly, the SLC3A2∗SLC7A11 and SLC3A1∗SLC7A9 heterodimers mediate cystine uptake in exchange for Glu export (36), suppling cysteine to glutathione (GSH) biosynthesis and mitigation of oxidative-induced cell death by ferroptosis or pyroptosis (37, 38, 39). SLC3A2 is a major ligand for Gal3 (40, 41), and the proximity afforded by Gal3 clustering of SLC3A2∗SLC7A exchangers with Na^+^/AA symporters may provide a means of regulating the functional interaction reported in these earlier studies (32, 35).

**Figure 1:**
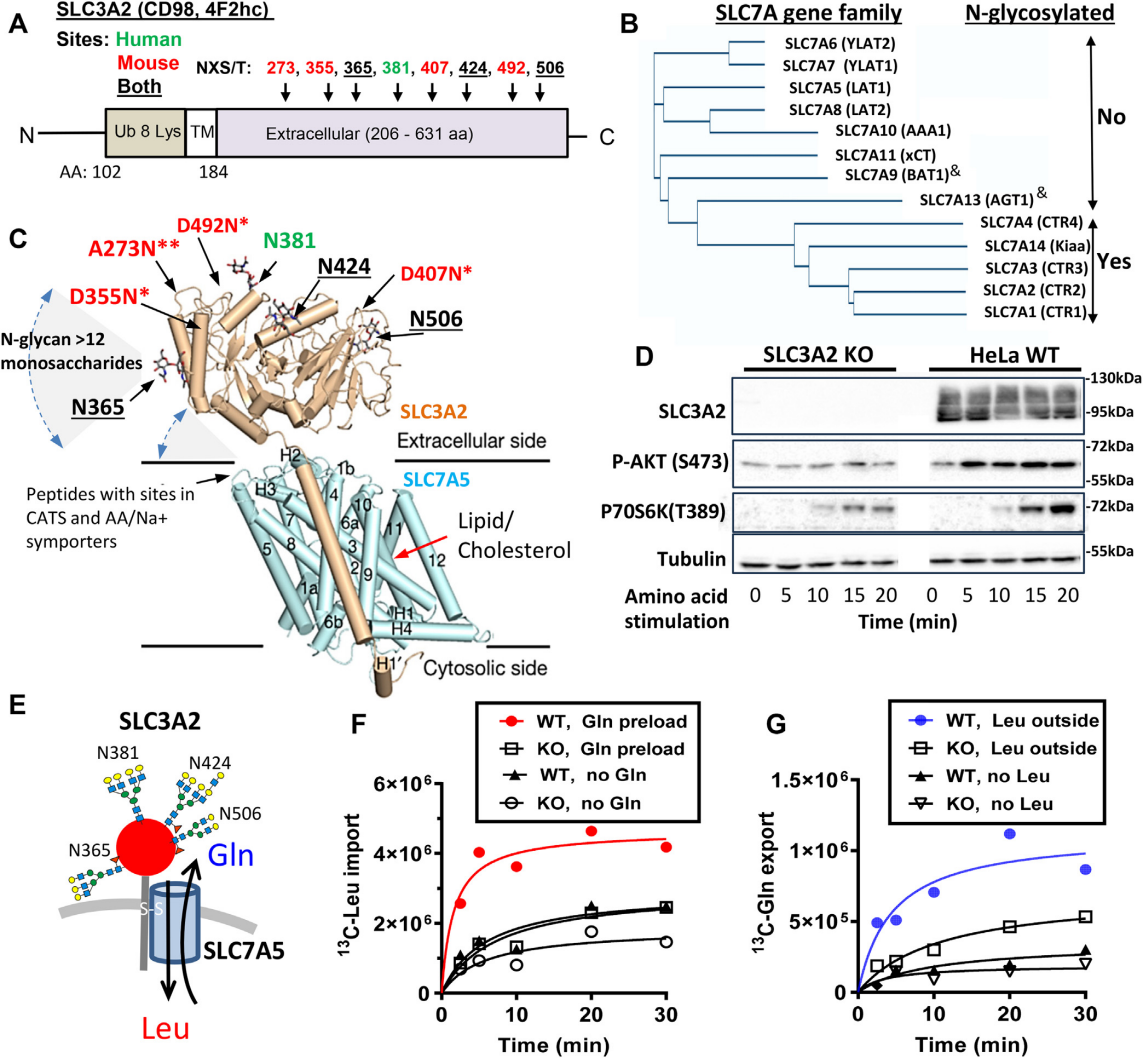
**SLC3A2 N-glycosylation sites.***A*, N-glycosylation sites NXS/T(X≠ P) in the human SLC3A2 sequence (*black, green*) and losses (*red*) since a common ancestor with mouse. Eight Lys residues near the N-terminus are targets for ubiquitination. *B*, phylogram of human SLC7A1-14 aligned by protein sequences and protein names are in brackets. Members of the family lacking N-glycosylation (No) dimerize with SLC3A2, or SLC3A1 marked by &. The N-glycosylated members (Yes) do not heterodimers with the adaptors. *C*, X-ray crystal structure of SLC3A2 in complex with SLC7A5 AA exchanger (50), and N-glycosylation sites with the linkage monosaccharide attached. The full-length N-glycans are commonly >12 monosaccharides and extend into solution with rotational flexibility about the glycosidic linkages. ∗ and ∗∗ indicate one or two mutations away from Asn for site lost since common ancestor with mouse. *D*, stimulation of mTOR kinase activity revealed by phosphorylation of p70S6 kinase in WT and SLC3A2 KO HeLa cells starved of amino acids for 1 h, followed by 15 min stimulation with Leu, Gln, Arg. *E*, schematic representing the major de-sialylated N-glycan structures at each site in SLC3A2 as documented herein. ∗ at N365, branching is a mix of bi-, tri- and tetra-antennary (see Fig. 2). *F* and *G*, SLC3A2 is required for Leu/Gln exchange activity. *F*, Time course of Leu import into AA starved HeLa cells, pretreated with ± 4 mM Gln for 1 h, washed and pulsed with 0.4 mM [U^13^C]-Leu in the medium. *G*, time course of Gln export from AA starved cells pretreated with 4 mM [U^13^C]-Gln for 1 h and pulsed with 0.4 mM ± Leu in the medium as per (32).

Cancer cells often over-express SLC3A2, SLC7A5, SLC7A11 (34) and N-glycans branching (42, 43), which support tumor progression by increasing mTOR signaling and mitigation of oxidative stress (ox-stress). For a subset of EAAs, the branched-chain amino acids (BCAA: Leu, Ile, Val), elevated serum levels are associated with insulin resistance and shortened lifespans (44, 45). Excessive weight gain on a high-fat diet is normalized by reducing BCAA in the diet without compromising lean tissue mass (44, 46). Herein, we have profiled SLC3A2 N-glycan structures at each site and analyzed their impact on trafficking, protein interactions, cellular AA balance, and sensitivity to ox-stress. Our results indicate that N-glycans at N381 and N365 interact with the Gal3 lattice, which promotes clustering between SLC3A2∗SLC7A5 and AA/Na^+^ symporters that regulate AA balance and GL-Lect mediates endocytosis. Curiously, SLC3A2 and associated transporters are absent in Neoaves (modern birds), indicating selection against this functional module on the path to the improved bioenergetics and longevity of many species (Ng, Pawling and Dennis accompanying manuscript (47)).

## Results

### SLC3A2 N-glycans: structures, trafficking, and protein interaction

The position and number of N-glycosylation sites on SLC3A2 have evolved extensively with the emergence of primates (Figs. 1*C* and S1, *A–C*). The consensus motif is Asn-X-Ser or Thr where X≠ Pro, (NXS/T(X≠ P)). Ancestral sites at four of eight positions vary locally by about 3 to 6 AAs over mammalian evolution, suggesting a conservative type of “experimentation” at 271 to 273, 355 to 352, 407 to 409, and 492 to 498. These sites were lost in primates and a novel site was acquired at N381. The other three sites at N365, N424, and N506 are conserved (Fig. S1*C*). NXS/T(X≠ P) displays an encoding asymmetry that accelerates experimentation with N-glycan positions (*i.e.*, two Asn and 10 Ser/Thr codons); a feature that enhances the evolvability of glycoproteins (48, 49). The four site losses in SLC3A2 were the more-likely non-synonymous mutations at the Asn. The N381 site gain created an Asn (DSS→ NSS) the less probable path but readily adopted by positive selection in primates. X-ray crystallography and cryo-electron microscopy of human SLC3A2 in complex with SLC7A5 has revealed interactions between the proteins on the extracellular side in close proximity to the membrane (50) (Fig. 1*C*). Selective pressures on SLC3A2 N-glycosylation sites have been greater than other adaptors to ion transporters with a similar heterodimeric structure. Notably, the Ca^++^ channel (ATP2B1) and Na^+^/K^+^ ATPase exchangers (ATP1A1) have highly conserved N-glycan positions in their adaptors, neuroplastin (NPTN) and ATPase subunit β1 (ATP1B1), respectively (51).

### Site-specific N-glycan branching on SLC3A2

The Flp-In T-REx HeLa cells (designated as WT cells below) were targeted for SLC3A2 deletion by CRISPR-Cas9. Mutant cells displayed a reduced capacity for mTOR activation by Leu (Fig. 1*D*), slower rates of growth and cell migration (Fig. S2, *A–C*); which is consistent with phenotypes previously reported for SLC3A2 KO cells (32, 52). ^13^C-Leu uptake and ^13^C-Gln export were impaired by ∼3 fold under maximal inside-to-outside gradient conditions, a marked reduction in EAA/Gln exchange activity by SLC7A5, as previously reported (32) (Fig. 1, *E*–*G*).

**Figure 2:**
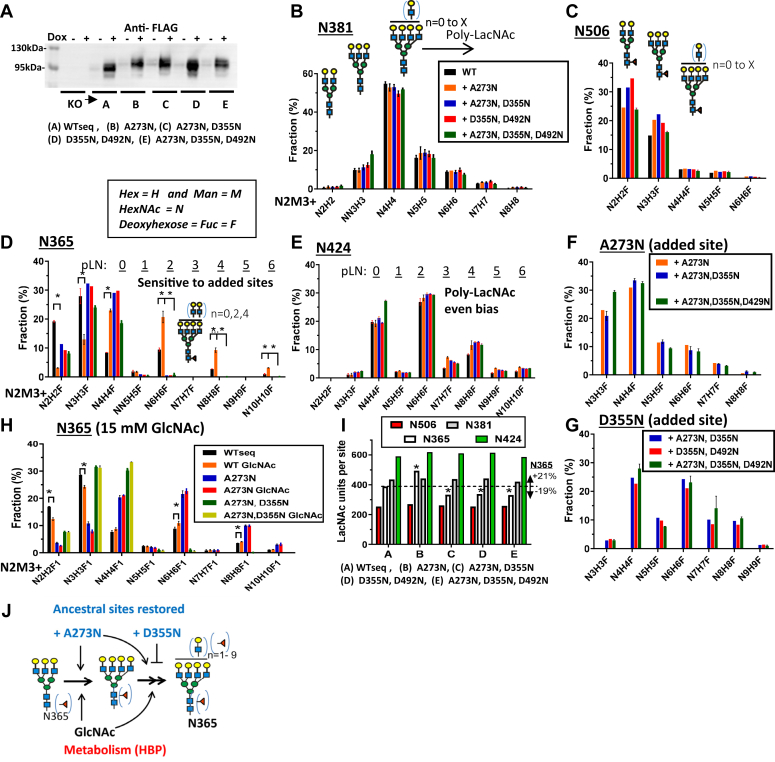
**Position-dependent remodeling of N-glycan on SLC3A2.***A*, SLC3A2 Western blot of anti-FLAG pulldowns from lysates of cells treated with and without dox. *B*, glycan structures at N381, (*C*) N506, (*D*) N365, (*E*) N424, and restored ancestral sites (*F*) A273N, (*G*) D355N. The site added at N492 was not glycosylated. Mean ± SD of three experiments by LC-MS/MS, each with three technical replicates. pLN refers to poly-LacNAc length. *H*, GlcNAc supplementation (48 h) increases poly-LacNAc content on tetra-antennary N-glycans at N365, causing reciprocal decrease in bi- and tri-antennary glycans ∗*p* < 0.05, n = 3 (see Fig. S7). *I*, N-acetyllactosamine (LacNAc) content at each site on FLAG-SLC3A2 in WTseq and gain-of-site variants. N365 LacNAc content is increased by 21% by adding A273N and reduced by 19% by D355N sites ∗ *p* < 0.05, n = 3. *J*, graphical summary of the positive and negative effects of added sites and GlcNAc supplementation on branching at N365.

To understand the utility of SLC3A2 N-glycans, we began by profiling the structures at each native site, and inserted sites where they occur in mice but are absent in primates (Fig. 1, *A* and *C*). An N-terminal FLAG-tagged SLC3A2 sequence (P08195-1), (abbreviated herein WTseq), and gain-of-site FLAG-tagged variants A273N, D355N, and D492N were inserted into the Flp-In site of SLC3A2 KO cells for expression under the control of a tetracycline (dox)-inducible promoter. FLAG-SLC3A2 variants displayed similar levels of dox-induced expression and slower mobility on gel electrophoresis consistent with additional N-glycosylation (Fig. 2*A*). Sites at A273N and D355N were N-glycosylated, and D492N was unoccupied based on LC-MS/MS analysis of the peptide sequence.

Each site in FLAG-SLC3A2 displayed distinct and reproducible profiles of post-Golgi-modified N-glycans (Fig. 2, *B*–*E* and Table S1). The tetra-antennary content was higher at N381 and N424 than the other sites, and at N424, the N-glycans were more frequently extended with linear poly-N-acetyllactosamine (poly-LacNAc) chains. N-glycans at N506 were mostly bi- and tri-antennary, while N365 displayed a more complex mix of bi-, tri-, and tetra-antennary some with poly-LacNAc. The total LacNAc content ranked by site was N424 > N381 ∼ N365 > N506. Endogenous SLC3A2 in HeLa cells displayed site-specific N-glycan profiles consistent with that of dox-induced FLAG-SLC3A2 (Fig. S3 and Table S2). Thus, the microenvironment of N-glycan sites influences the access and cooperativity or interference between enzymes in the Golgi remodeling pathway (53). Further LC-MS/MS fragmentation of the N-glycans provided additional information (Fig. S4, *A–F*). We observed a remarkable bias toward numerically even LacNAc units in poly-LacNAc chains at N424, N365, and the inserted site D355N, while N-glycans at N381 displayed the expected second order decay in elongated poly-LacNAc chains (Fig. 2, *B*–*G*). At the N365 site only, poly-LacNAc content was increased with the addition of A273N, while the co-addition of D355N suppressed the enhancing effect of A273N (Fig. 2, *D* and *I*). Importantly, the N-glycan added at D355N completely suppressed the A273N enhancement and the WTseq levels of poly-LacNAc at N365. N-glycan profiles for N424 and N381 were not significantly affected by the presence of the added sites. N365 was also the only site displaying reduced bi-, tri-, and increased tetra-branched N-glycans with poly-LacNAc when the cultures were supplemented with GlcNAc (Figs. 2*H* and S5 and Table S3). The unique microenvironment of N365 is an example where neighboring sites and HBP metabolism are factors in the regulation of N-glycan processing (Fig. 2*I*). The results suggest that site losses in SLC3A2 on the evolutionary path to primates have shifted regulation at N365 away from neighboring sites and toward a dependence on HBP (Fig. 2*J*).

### Site-specific regulation of endocytosis and surface retention by galectin 3

SLC3A2 is both a positive and negative regulator of T-cell proliferation (54, 55). As a positive regulator, the extracellular domain of SLC3A2 projects branched N-glycans at N381 and N424 above the membrane by >100 Å where interaction with the Gal3 lattice may promote surface retention as previously observed for receptor kinases (10). As a negative regulator, the N-glycan at N365 is close to the membrane (∼10 Å) (Fig. 1*C*), where Gal3 binding in more likely to promote GL-Lect-driven endocytosis and trafficking to recycling endosomes or ubiquitination and lysosomal degradation (23).

**Figure 3:**
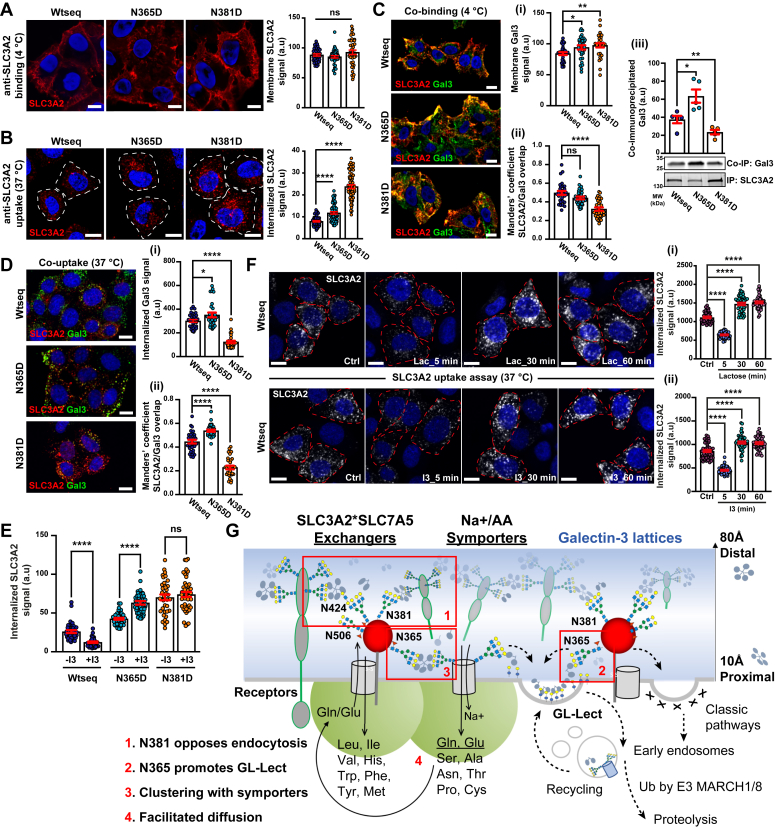
**N-glycans and SLC3A2 endocytosis.***A*, surface levels of FLAG-WTseq and variants are similar. Cells were incubated for 30 min at 4 °C with 10 μg/ml of anti-SLC3A2 antibody (*red*), nuclei by DAPI (*blue*). Unbound antibody was removed with ice-cold PBS, cells fixed with 4% PFA, and immunolabeled. The SLC3A2 signal was quantified (*right histogram*). Means ± SEM, one-way ANOVA; NS, non-significant. Scale bars = 10 μm. *B*, internalization of SLC3A2 site variants is increased. Cells were continuously incubated for 10 min at 37 °C with 10 μg/ml anti-SLC3A2 antibody (primary antibody), and then shifted to 4 °C. Cell surface-accessible antibody was removed on ice with acid wash solution. Cells were then fixed, permeabilized with saponin, immunolabeled, and images were quantified (right histogram). Means ± SEM, one-way ANOVA; ∗*p* < 0.05, ∗∗*p* < 0.002, ∗∗∗∗*p* < 0.0001. Scale bars = 10 μm and in all panels. *C*, co-distribution of SLC3A2 and Gal3. Cells were incubated for 30 min at 4 °C with 5 μg/ml of purified Gal3-488, and after washing for 30 min at 4 °C with 10 μg/ml anti-SLC3A2 antibody (primary antibody). After extensive washes with ice-cold PBS, cells were fixed and immunolabeled with secondary antibody. Gal3 signal at the membrane and its colocalization with SLC3A2 were quantified in the `histograms. (i) Surface levels of Gal3 are similar; (ii) Mander’s coefficient dropped significantly for the N381D variant; (iii) co-immunoprecipitation with Gal3 was increased for N365D and decreased for N381D. *D*, same as in (C) with the additional shift for 10 min at 37 °C for measuring internalization. (i) Endocytosis of Gal3 is slightly increased in N365D expressing cells and strongly decreased in N381D expressing cells; (ii) Mander’s colocalization coefficient of Gal3 with SLC3A2 is increased in N365D expressing cells and strongly decreased in N381D expressing cells. *E*, Gal3 inhibition and SLC3A2 endocytosis. Cells were preincubated for 5 min at 37 °C with 10 μM of Gal3 inhibitor GB0149-03 (termed I3 in this figure). I3 solution was removed, and cells were continuously incubated for 10 min at 37 °C with 10 μg/ml of anti-SLC3A2 antibody. Internalized SLC3A2 signal was quantified. *F*, comparison of acute *versus* prolonged galectin inhibition on SLC3A2 uptake. Cells expressing the SLC3A2WTseq cells were pretreated for 5 (acute), 30 or 60 min (prolonged) with either (i) the general galectin inhibitor lactose, or (ii) the Gal3-specific inhibitor I3 (see Experimental procedures), followed by anti-SLC3A2 antibody uptake assay (see Experimental procedures). Internalized SLC3A2 signal intensities were quantified in both I3 and lactose conditions (histograms). With both inhibitors, acute treatment (5 min) induced a significant inhibition of SLC3A2 endocytosis, whereas SLC3A2 uptake was increased with the prolonged treatment schedules. A single median plane from confocal imaging was represented. *Red dashed lines* represent the cell contours. *G*, working model. Surface expression (1), endocytic trafficking (2), interactions of SLC3A2∗SLC7A5/11 (3), and lateral diffusion (4) regulated by Gal3 are highlighted in *red*.

To test the hypothesis that SLC3A2 N-glycans link membrane proximal and distal interactions, dox-induced FLAG-SLC3A2 WTseq, N381D, and N365D site variants were tracked in live cells by immunocytochemistry. The levels of cell surface FLAG-SLC3A2 were similar for WTseq, N381D, and N365D site variants after 48 h of dox induction as evidenced by anti-SLC3A2 antibody binding on ice (Fig. 3*A*). However, upon incubation at 37 °C for 10 min, endocytosis of FLAG-SLC3A2 was strongly increased for N381D and intermediate for the N365D site variant relative to the WTseq, indicating an N-glycan position-dependent effect (Fig. 3*B*). Internalized N381D was primarily localized to large perinuclear endosomes, a phenotype also seen in cells expressing the N365D variant although less pronounced. In contrast, internalized FLAG-SLC3A2 WTseq remained mostly peripheral in small discrete endosomes characteristic of recycling endosomes (Fig. 3*B*). The results suggest that the primate-derived configuration of four N-glycans opposes endocytosis, and deletion of N-glycans at N365 and N381 affected cellular entry and trafficking in different ways.

Next, we asked whether N-glycan positions on SLC3A2 influence Gal3 binding. Fluorophore-tagged Gal3-488 and anti-SLC3A2 were sequentially incubated at 4 °C with WTseq or site variant expressing cells, which revealed a small increase in Gal3 at the surfaces of cells expressing the variants (Fig. 3*C* (i)). The Manders’ coefficient for SLC3A2 – Gal3 colocalization remained similar for the N365D variant, it was significantly decreased in N381D expressing cells (Fig. 3*C* (ii)) consistent with loss of a higher affinity tetra-antennary Gal3-binding N-glycans positioned away from the membrane (Figs. 1*C* and 2*B*). Correspondingly, surface-bound anti-SLC3A2 antibodies pulled-down less Gal3-488 from lysates of N381D expressing cells, when compared to WTseq, and more from N365D lysates where the N-glycan at N381 is likely more exposed to the Gal3 lattice (Fig. 3*C* (iii)). Cells that had sequentially bound Gal3-488 and anti-SLC3A2 were then shifted to 37 °C for 10 min to assess the co-uptake. Gal3-488 uptake was decreased with N381D, when compared to the WTseq, while it was slightly increased with N365D (Fig. 3*D* (i)), which mirrored the co-immunoprecipitation data in Figure 3*C* (iii). Furthermore, internalized Gal3 and SLC3A2 colocalized strongly in the N365D compared to N381D expressing cells (Fig. 3*D* (ii)). Perhaps loss at N365D, the site nearest the membrane, reduces Gal3-mediated cross-linking of this variant with glycolipids, thereby reducing competition for Gal3 binding to N381 and N424.

The data is consistent with the N-glycan at N381 acting as a high-capacity interaction site for the Gal3 lattice, while the effects of the N-glycan at site N365 appear to be more complex. To explore this idea further, cells were pre-incubated (5 min) with a Gal3 inhibitor GB0149-03 (56) in the same assay protocol. For WTseq-expressing cells, GB0149-03 inhibited SLC3A2 internalization by ∼50% (Fig. 3*E*), indicating a substantial portion mediated by Gal3-dependent GL-Lect, in agreement with an earlier report that identified SLC3A2 as a cargo for clathrin-independent endocytosis (57). In contrast, GB0149-03 treatment increased endocytosis of the N365D variant and had no additional effect on the already higher amount of internalized N381D variant (Fig. 3*E*). The results support the hypothesis that the N-glycans far from the membrane at N381 has a major role in surface retention by the Gal3 lattice, while the proximity of the N365 N-glycan to glycolipid-enriched domains promotes GL-Lect-driven endocytosis. The N365 N-glycan also contributes to surface retention, as NXS/T site multiplicity and N-glycans branching are additive factors in glycoprotein affinity for the Gal3 lattice (58).

As expected, weaker binding of site-loss variants to the Gal3 lattice accelerated their internalization by clathrin-dependent endocytosis, caveolae (10, 12, 14) or micropinocytosis (21), thereby masking or subverting the GL-Lect driven process. To test this hypothesis more directly, we compared the endocytic fate of SLC3A2 WTseq following acute GB0149-03 or lactose pretreatment for 5 min compared to extended preincubations with these compounds for 30 or 60 min. As already described in Figure 3*E*, the acute 5-min inhibitor preincubation led to ∼50% inhibition of SLC3A2 WTseq endocytosis, whereas by 30 or 60 min, endocytosis was increased well above that of the untreated control (Fig. 3*F*). As with the N365D and N381D variants, extended inhibitor treatment drove a significant fraction of the SLC3A2 WTseq into perinuclear endosomes in which SLC3A2 WTseq overlapped more strongly with the early endosomal marker EEA1 (Fig. S6*A*). The results suggest that the four occupied sites on WTseq secure SLC3A2 to the Gal3 lattice and allow us to observe rapid GL-Lect mediated endocytosis as a GB0149-03 or lactose inhibitable process at 5 min of pretreatment, whereas at 30 to 60 min, endocytosis by coated-pits and caveolae pathways dominate (10, 11, 12, 14) (Fig. 3*F*). The weaker affinities of N381D and N365D variants at 5 min enhance internalization by these classic pathways and mask GL-Lect-mediated endocytosis (Fig. 3*E*). Taken together, surface retention by the Gal3 lattice and GL-Lect driven endocytosis appear to be opposing forces on SLC3A2 trafficking, balanced by the evolved multiplicity and positions of N-glycan on the extracellular fold (Fig. 3*G*).

### SLC3A2 protein interactome

**Figure 4:**
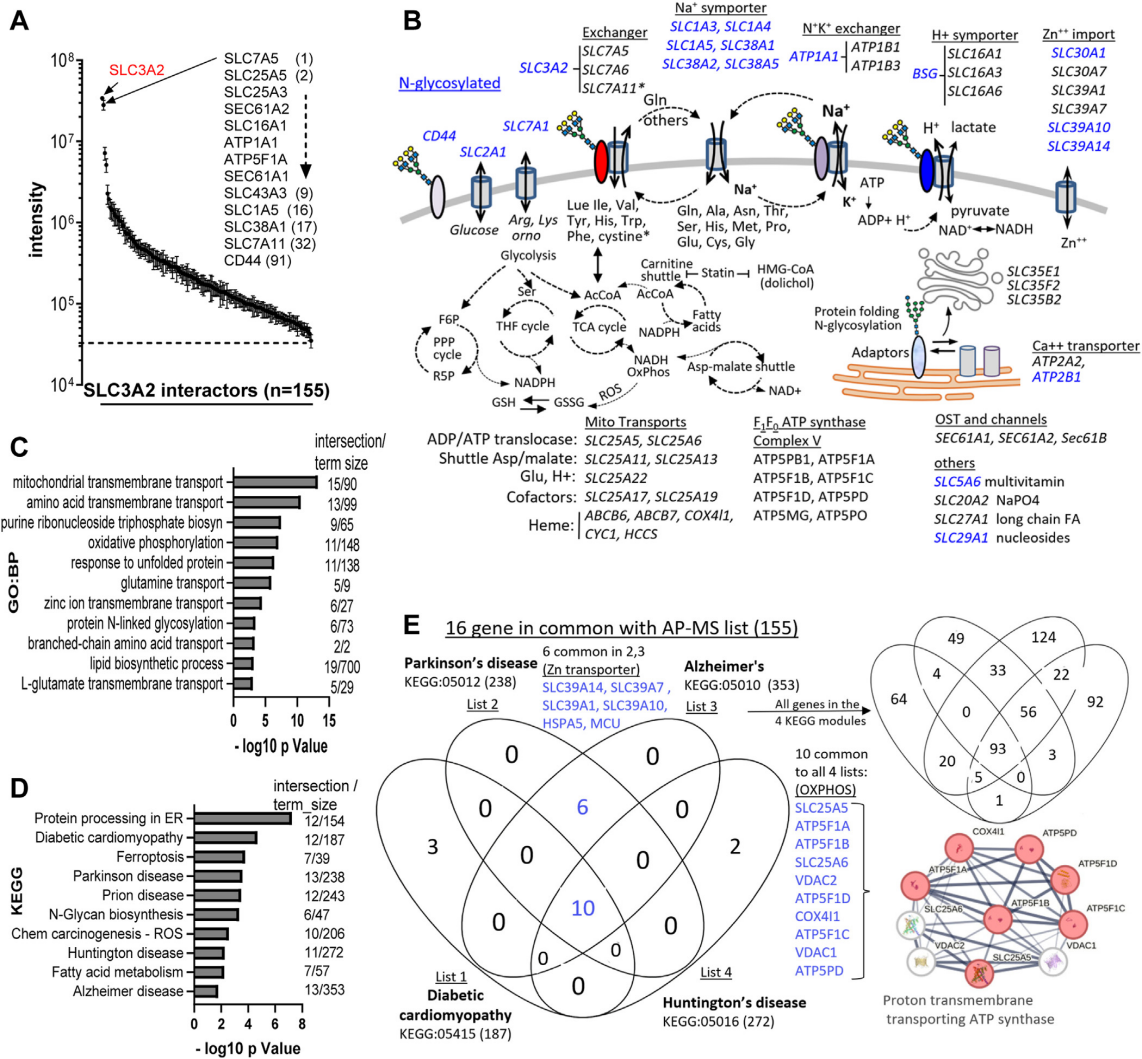
**SLC3A2 interacting proteins.***A*, AP-MS with anti-FLAG antibodies in dox-induced FLAG-SLC3A2 cells revealed peptides from 155 interacting proteins with mass intensities >3 SD above control pulldowns from dox-treated SLC3A2 KO cell lysates (mean ± SD, n = 5 experiments). The results are plotted in rank order of peptide intensity. *B*, schematic of SLC3A2 interacting proteins associated with AA and ion gradients impacting metabolism. N-glycosylated proteins are marked in *blue*. *C*, gene ontogeny Biological Process and (*D*) KEGG by gProfiler using ordered query for the 155 FLAG-SLC3A2 interacting proteins. *E*, sixteen SLC3A2 interacting proteins (*blue*) are associated with four degenerative diseases, which segregate into two functional classes (Venny Diagram, J.C. Oliveros). The STRING network represents the 10 transmembrane protein transporters. Four SLC39A transporters regulate zinc import/export.

Affinity purification-mass spectrometry (AP-MS) with dox-induced FLAG-SLC3A2 WTseq as bait identified 155 significantly enriched proteins (Fig. 4*A* and Table S4). Approximately 75% of SLC3A2 is disulfide-linked in HeLa cells (Fig. S6*B*), and in this category, SLC7A5 was by far the top interactor with SLC7A11 in 32nd position (Fig. 4*A*). Dox-induced FLAG-SLC3A2 rescued the stress-response and resistance to H_2_O_2_, indicating that the protein interactions observed were that of a functional bait (Fig. S7, *A* and *B*). Dox-induced expression and pull-down efficiencies were similar for Wtseq and variants (Fig. S7*C*). By gene ontology (V11.0), the list of interactors included 115 integral components of membranes, of which 70 were nutrient and ion transporters, including Aas, monocarboxylate, ATPase Na^+^/K^+^ exchangers, and Zn^++^ transporters (Fig. 4*B*). The set of 155 proteins also included eight complex V ATP synthases (ATP5 family), ADP/ATP translocator (SLC25A5, SLC25A6), Asp/Malate and αKG/malate shuttle (SLC25A11), thiamine pyrophosphate transport (SLC25A19) and Glu transporters (SLC25A22, SLC25A13, SLC25A11). Other novel interactors included a mitochondrial transmembrane transport complex; purine ribonucleotide biosynthesis, protein N-glycosylation, and lipid biosynthesis-associated proteins (Fig. 4, *B* and *C* and Table S4). GO:KEGG points to diseases associated with oxidative and mitochondrial stress (Huntington, Parkinson, Alzheimer, Diabetic cardiomyopathy) (Fig. 4, *D* and *E*). Importantly, SLC3A2 interacted with other glycoprotein adaptors; CD44, BSG, ATP1B1, and ATP1B3, which partner with non-glycosylated transporters that are also regulated by clathrin-independent carriers (57, 59) (Fig. 4*B*).

### SLC3A2 interactions dependent on N-glycans

**Figure 5:**
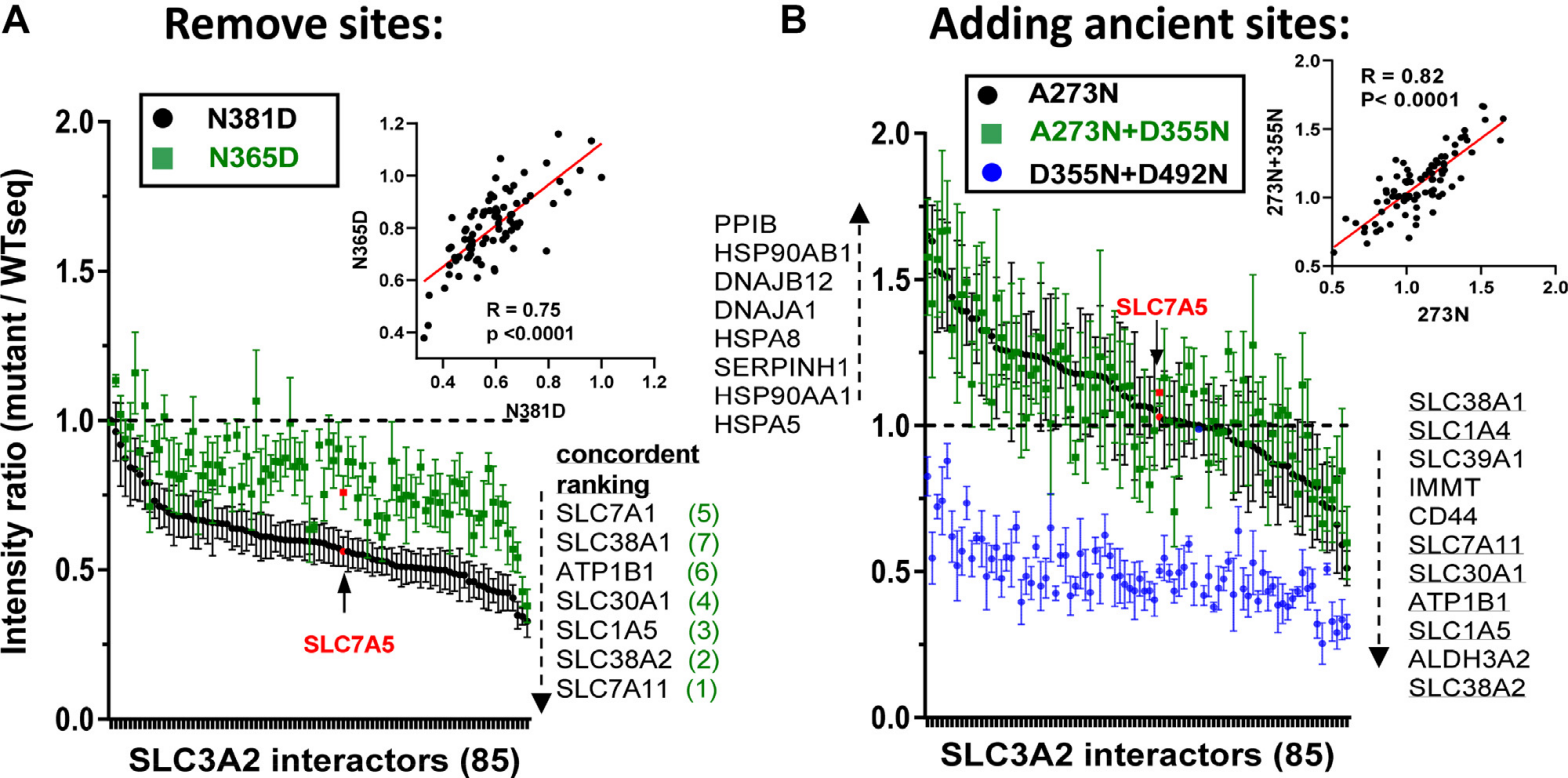
**Protein interactions with SLC3A2 depend on N-glycosylation.***A*, AP-MS analysis of anti-FLAG antibody pulldowns from dox-induced FLAG-SLC3A2 WTseq and site-deletion variants, shown as mutant/WTseq ratios, mean ± SE, WTseq, N381D (n= 8 each). Data is graphed relative to the ranking of N381D/WTseq. *B*, ratios from WTseq n = 4, A273N n = 3, A273N + D355N n = 3, and D355N + D492N n = 3 independent pulldowns. Graphed relative to the ranking of A273N/WTseq. Inserts show correlations between variant pairs. For the variant in *blue*, the added site at D355N was glycosylated and D492N was not.

We focused on 85 of the 155 SLC3A2 interactors by AP-MS that displayed significant differences between consensus and site variants in a Kruskal Wallis test of all and pairwise comparisons. With N381D and N365D site mutations, FLAG-SLC3A2 interactions decreased including SLC7A5 by 44 ± 14% and SLC7A11 by 67 ± 15% as well as neutral AA/Na^+^ symporters SLC1A5, SLC1A4, SLC38A2 and SLC38A1; Arg/Lys/Ornithine high-affinity permease SLC7A1, the Zn^++^ transporter SLC30A1, and non-catalytic subunit of Na^+^/K^+^ ATPase exchanger ATP1B1 (Fig. 5*A* and Table S4).

SLC3A2 with the A273N and A273N +D355N insertions increased SLC3A2 association with ER chaperones (*i.e.*, HSPA5, HSP90AB1, HSP90AA1, and HSPA8) indicating ER stress (Fig. 5*B* and Table S4). These sites-added variants reduced the association with transporters that we observed above with the site deletion variants (*i.e.*, SLC38A2, SLC1A5, ATP1B1, SLC30A1 SLC7A11, SLC39A1, SLC1A4, and SLC38A1) (Figs. 4*A* and 5*B*), suggesting a greater sensitivity to the status of SLC3A2 N-glycosylation. N-glycosylation at D355N suppressed SLC3A2 interactions widely, perhaps due to a greater negative impact of D355N on poly LacNAc content or an uncharacterized impact of the D492N mutation (Fig. 2, *I* and *J*).

SLC38A2, SLC1A5, SLC30A1, SLC1A4, SLC38A1 and SLC7A1 are N-glycosylated, and have also been identified as prey that correlate with SLC3A2 in a global BioID proteomics network (60). The BioID method depends on proximity over time rather than affinity and can therefore detect weak multivalent interactions of proteins with intrinsically disordered domains that form stress granules (61). Similarly, the glycosidic bonds of N-glycan chains allow for an inherent disorder and give way to stochastic multivalency binding within the planar Gal-3 lattice on the membrane (22). The avidity is regulated by branching and metabolic flux to HBP. Experimental and inferred interactions between SLC7A exchangers and AA/Na^+^ symporters occur frequently in the literature, perhaps nearer the affinity cut-offs, and are infrequently selected for follow-up in most proteomics studies.

### Evidence for functional interactions between EAA exchanger and Na^+^/AA symporters

**Figure 6:**
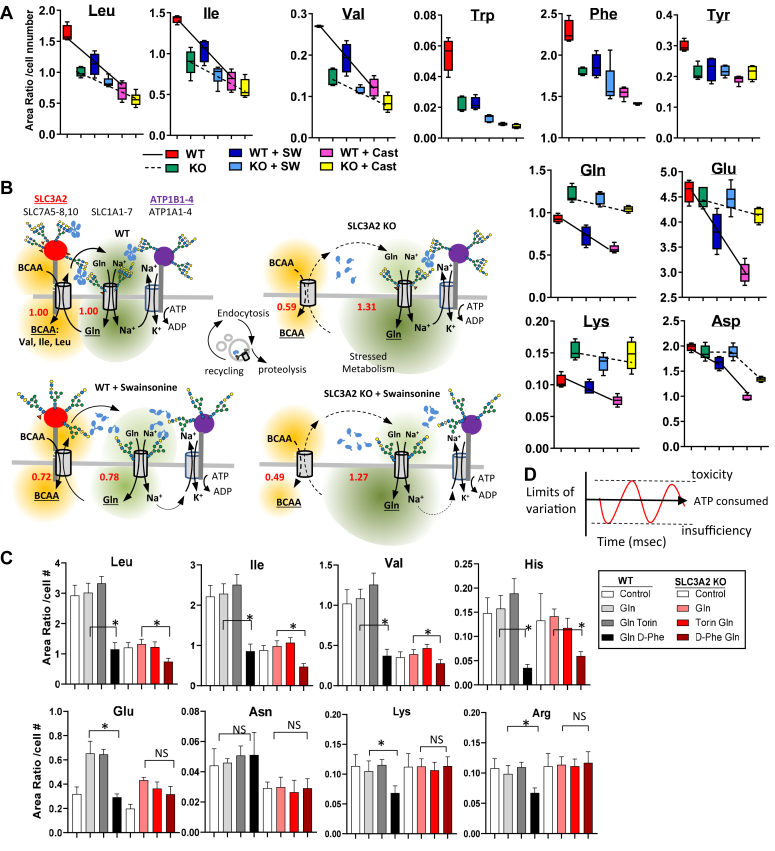
**N-glycan branching promotes processivity between AA exchanger and symporters.** WT and SLC3A2 KO HeLa were grown in DMEM + 10% FCS with and without 250 ng/ml swainsonine or 10 mg/ml castospermine for 48 h, then prepared for metabolite extraction and analysis by LC-MS/MS (Table S5 and Fig. S8). *A*, intracellular amino acids level. *B*, an interpretive model. Numbers in *red* are changes in BCAA/Gln ratio relative to WT untreated (ratios for Leu, Ile, Val were averaged). BCAA/Gln in swainsonine treated WT are balanced while SLC3A2 KO cells remains imbalanced. *C*, metabolite levels in WT and SLC3A2 KO cells grown in DMEM + 10% FCS without Gln for 16 h (control) then supplemented with Gln (2 mM), Torin (250 nM) and D-phenylalanine (50 mM) in the final hour as indicated. *D*, the energy required to limit variation in EAA is expected to be proportional to AA transfer rates and frequency of oscillations driven by intracellular metabolism.

Clustering of SLC3A2∗SLC7A exchangers with Na^+^/AA symporters (SLC1A and SLC38A families) by Gal3 crosslinking may facilitate EAA exchange and recovery of Gln. Metabolite release and exchange between cells in tissues is a means of coordinating metabolism and signaling in tissues (62, 63, 64). If branched N-glycans on SLC3A2 play a role, inhibitors of the pathway may interact with the SLC3A2 KO phenotype in an informative manner. Treatment with swainsonine (SW), an α-mannosidase II inhibitor, reduced bi, tri, and tetra branched N-glycan by ∼90% leaving mature N-glycans with only one branch; the product of MGAT1 (Fig. S8, *A* and *B*). In WT cells, SW reduced EAA as well as Gln, Glu, and Lys in a balanced manner by ∼25%. In untreated SLC3A2 KO cells, EAA levels were suppressed (0.59) and Gln was elevated (1.31). The imbalance was maintained in SW-treated SLC3A2 KO cells (Fig. 6, *A* and *B* and Table S5). The results suggest that SLC3A2 KO has a major disruptive effect on the capacity of EAA/Gln exchangers to maintain AA balance, and as SW reduces affinities for Gal3 globally, interactions and balance can be maintained at lower AA levels (Fig. 6*B*). Castanospermine, an inhibitor of endoplasmic reticulum α-glucosidase II removes all the branches and reduces AA levels further than SW. Substrates of HBP (glucose, Gln, Acetyl-CoA) and UDP-GlcNAc were elevated in SLC3A2 KO cells (Fig. S8, *C* and *D*); positive feedback directed at rebalancing EAA/Gln/Glu by increasing N-glycan branching on SLC3A2 and interacting glycoproteins (65). An incomplete or delayed rescue of metabolism by the dox-induced SLC3A2 variant N381D may explain the increase in Gal3 binding observed in Fig 3*C*i.

Treating WT cells with D-phenylalanine (D-Phe), an inhibitor of SLC7A5, reduced intracellular Leu, Ile, Val, His by ∼70%; levels comparable to that of untreated SLC3A2 KO cells (Fig. 6*C*). D-Phe reduced levels in SLC3A2 KO cells by a further ∼50%, consistent with a residual transporter activity in SLC3A2 KO cells that sustains growth (66). Gln levels were maintained in WT and KO cells when cultured in Gln-free medium for 16 h. Restoring Gln to the culture medium for one-hour increased Glu levels in WT cells, which was suppressed by the co-addition of D-Phe, indicating that Gln uptake and consequently Glu levels are dependent on SLC7A5 exchanger activity. However, D-Phe had no additional effect on Gln-dependent restoration of Glu levels in SLC3A2 KO cells (Fig. 6*C*). Thus, SLC3A2 is required in the cooperative interaction between SLC7A5 and Na^+^/AA symporters that balance EAA and Glu levels. Lateral diffusion of membrane-embedded proteins is slower by orders of magnitude than diffusion of AA in solution (67, 68). The results suggest that Gal3 mediated clustering of SLC3A2∗SLC7A5 and AA/Na^+^ symporters allow a processive exchange of EAA and efficient recovery of Gln as per Fick’s laws of diffusion.

**Figure 7:**
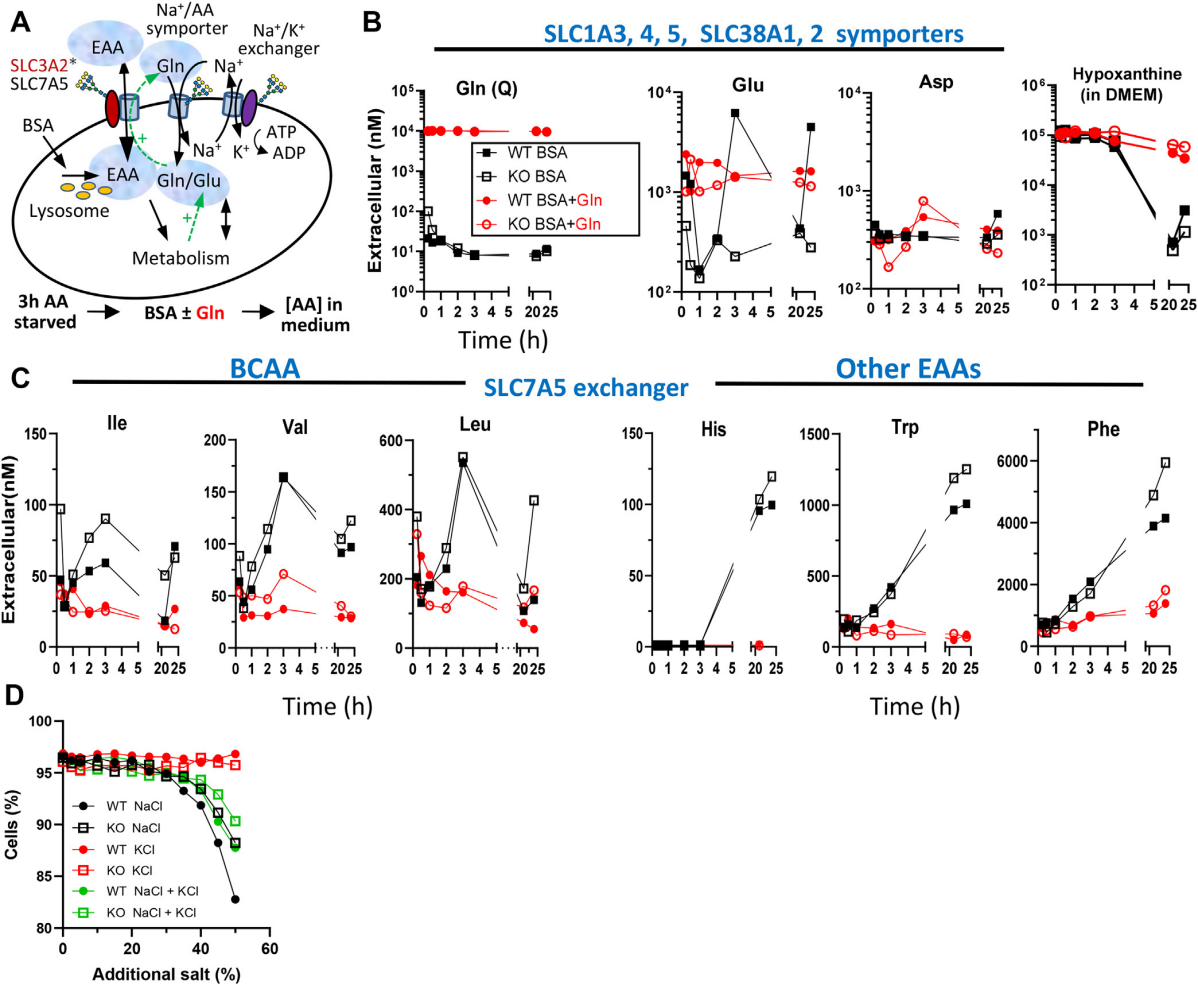
**Amino acid exchange between cells and culture medium.***A*, experimental scheme for AA derived from proteolysis of bovine serum albumin (BSA) and released into the medium. WT and SLC3A2 KO HeLa cells (6 × 10^5^ in 2 ml) were grown in AA-free medium for 3 h followed by a change of DMEM/F12 medium and supplementation with 3% BSA alone or + 2 mM Gln (BSA+Gln), and metabolites were measured in the medium by LC-MS/MS at times thereafter. *B*, BCAA (*C*) examples of other EAAs. Hypoxanthine was supplied in the AA-free medium, and salvaged by cells in BSA alone, and much less with Gln indicating that purine biosynthesis is active. *D*, NaCl, KCl, and a 1:1 mixture was added to the culture medium, and growth monitored 24 h later by Alamar Blue. SLC3A2 KO cells are more resistant to NaCl, suggesting a weaker interaction between SLC7A5 exchanger, Na^+^/AA symporter.

Processivity (limiting loss to diffusion) may be observed as synchrony of EAA and Gln/Glu exchange across the membrane. To explore this possibility, cells were conditioned in an AA-free medium for 3 h, followed by a change of the AA-free medium plus either Bovine Serum Albumin (BSA) or BSA+Gln, and extracellular AAs were measured at times thereafter (Fig. 7*A*). Phagocytosis of BSA and proteolysis in the lysosomes supply intracellular AAs and the ensuing imbalance is reflected in AA released to the medium (35, 69, 70). During the 3 h AA-fast, WT and mutant Hela cells released 15 of the 19 AAs measured, while Cys, His, Arg, and Met were absent in the SLC3A2 KO cell medium, and Lys was reduced (Fig. S9, *A* and *B*). With the addition of BSA alone, all AAs were present in the medium by 21 h except for Met. Gln levels declined rapidly to a new equilibrium by 2 h in WT and SLC3A2 KO cell medium. In contrast, Glu levels fluctuated in the medium of WT indicating a reversibility of transport direction by the cell population, which was lacking in SLC3A2 KO cultures (Fig. 7*B*). Importantly, Leu, Ile, and Val also showed a reversal of transport direction in synchrony with Glu levels in the WT cell medium (Fig. 7*C*). In contrast, Trp, Phe, His, Arg, and Lys increased continuously, reaching higher levels in SLC3A2 KO than in WT medium by 21 and 24 h. The initial fluctuations (0–3 h) in Glu and BCAA were suppressed after 3 h, by which time increasing Trp, Phe and His may act as counter-ions for BCAA import (71). Rates of metabolism in WT and SLC3A2 KO cells in BSA alone were similar as indicated by the consumption of hypoxanthine; a component of the AA-free medium (Fig. 7*B*). In BSA+Gln, the release of BCAA as well as Trp, Phe, His, Arg, and Lys from WT and KO cells was largely suppressed, suggesting an increased metabolic demand for EAA (Fig. 7*C*).

The results in BSA alone are consistent with processivity between SLC3A2∗SLC7A5 and AA/Na^+^ symporters that facilitate BCAA and Gln exchange. Coupling BCAA/Gln exchange with AA/Na^+^ import is expected to increase the demand for ATPase-dependent Na^+^/K^+^ exchange. Indeed, WT cells are more sensitive to increasing NaCl concentrations than SLC3A2 KO cells where coupling is impaired (Fig. 7*D*). Controlling the levels of BCAA may be more costly than other EAA as suggested by the reversal in transport direction by cells in BSA alone (Fig. 7, *B* and *C*). Exchanger-symporter proximity may maintain BCAA within limits that avoid adverse effects, but with a significant energy expenditure on BCAA/Gln cycling (Fig. 6*D*).

### Chronic stress and N-glycan processing in SLC3A2 KO cells

**Figure 8:**
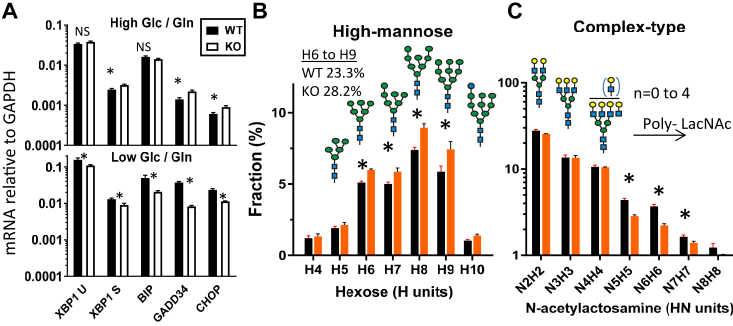
**SLC3A2 mitigates ER-stress under nutrient-limiting conditions.***A*, qPCR analysis for stress-inducible genes; XBP1, XBP1 splice, BIP, GADD34 and CHOP. WT and SLC3A2 KO HeLa cells cultures in high glucose/glutamine (25 mM/4 mM) or transferred to low (10 mM/1 mM) DMEM for 24 h. ∗*p* <0.01 paired *t* test. *B* and *C*, high-mannose and complex-type N-glycans with poly N-acetyllactosamine, released from WT and SLC3A2 KO cell membranes by PNGase F and analyzed by LC-MS/MS; Mean ± SD, ∗*p* <0.05 paired *t* test; three experiments with 2 to 3 technical replicates (Table S6). Galactose (*yellow circle*), GlcNAc (*blue square*), mannose (*green circle*).

SLC3A2 KO cells in normal culture conditions high glucose/Gln (25 mM/4 mM) displayed a small increase in ER stress-response transcripts *XBP1*, *HSPA5* (HSP70/BIP), *GADD34* and *CHOP* (Fig. 8*A* upper), which promote expression of antioxidant genes, including SLC3A2, SLC7A11, SLC7A1 and SLC1A5 (36, 72). However, transfer to lower glucose/Gln (10 mM/1 mM) medium stimulated expression of the ER stress-response genes in WT cells, but significantly less in SLC3A2 KO cells (Fig. 8*A* lower).

UDP-GlcNAc levels were strikingly elevated in SLC3A2 KO cells (Fig. S8*D*); perhaps as a feedback response to the AA imbalance that would normally strengthen transporter residency in the Gal3 lattice (65). However, an analysis of total cellular N-glycans revealed lower levels of complex-type N-glycans and more high-mannose structures [Man(6–9)GlcNAc2] in SLC3A2 KO cells, consistent with a chronic increase in misfolded glycoproteins (Fig. 8, *B* and *C* and Table S6). The removal of glucose residues from the high-mannose N-glycans added by oligosaccharyltransferase allows newly synthesized glycoproteins to enter the glucosidaseI/II Cnx/Crt cycle, an ancient chaperone and quality control system in the ER (3, 73). If folding fails after multiple cycles, the misfolded glycoprotein is targeted for ER-associated protein degradation (ERAD) (74). Poly-LacNAc extensions to branched N-glycans by *trans*-Golgi β1-3 N-acetylglucosaminyltransferases were also reduced, perhaps reflecting stress in the Golgi as well (18). In cell culture, titration with increasing H_2_O_2_ revealed an early transient increase in the Alamar Blue signal (*i.e.*, NAD(P)H/viable cell) in WT cells, indicating an induced resistance, which was markedly reduced in SLC3A2 KO (Figs. S7*B* and 9, *C* and *D*). Next, we explored stressed conditions, where SLC3A2 recruits CD44 and reorganizes the associated transporters to oppose stress (75, 76).

### Reduced NXS/T site occupancy with ER stress recruits CD44

**Figure 9:**
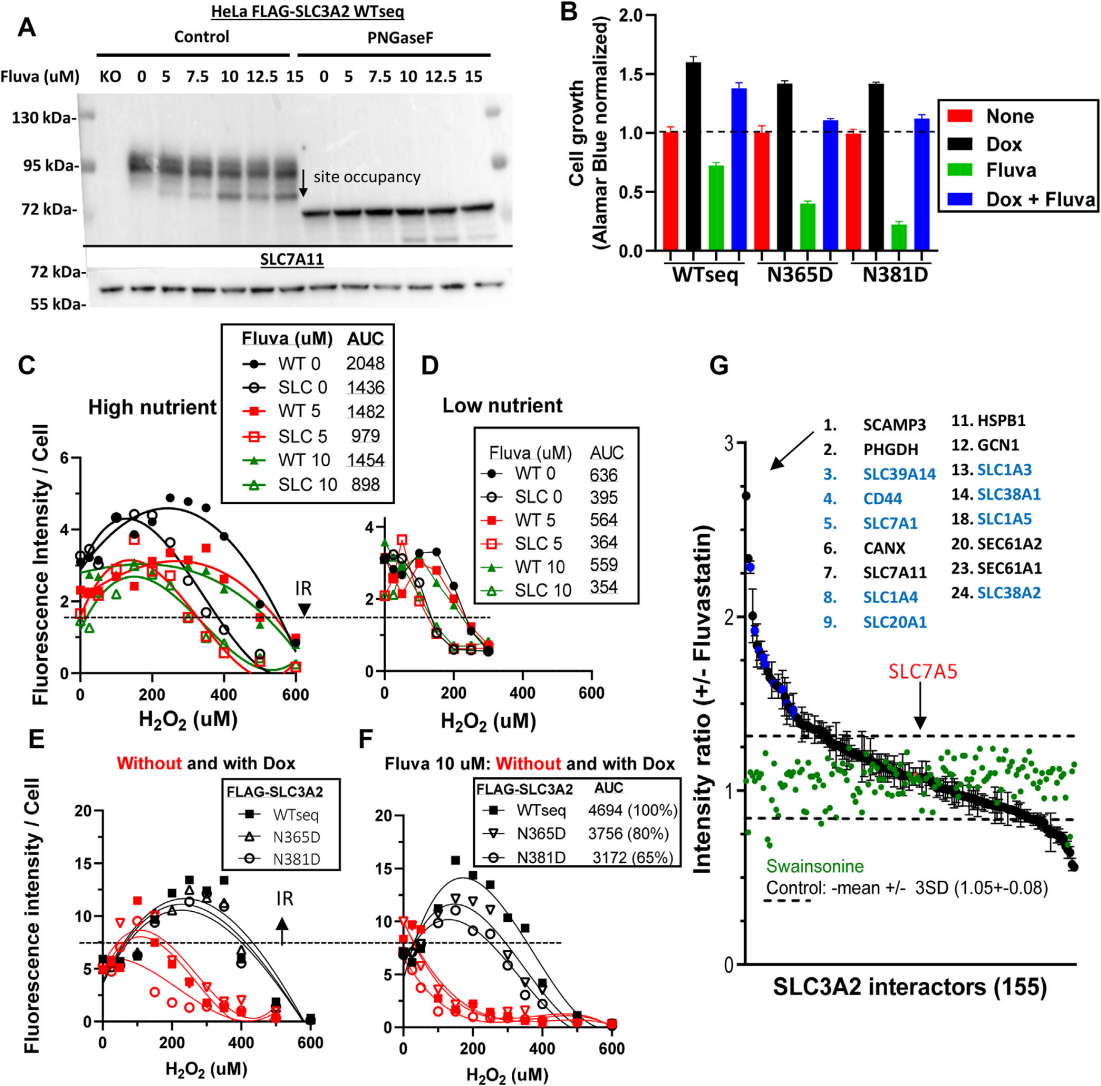
**SLC3A2 interactions in Fluvastatin-stressed cells.***A*, Western blots for dox-induced FLAG-SLC3A2 (WTseq) and SLC7A11 in lysates from HeLa cells, pretreated for 48 h with fluvastatin. Aliquots of lysate were treated with PNGase F (*right*). LC-MS/MS quantitation of unoccupied sites at N365, N381 and N424 in Fig. S9*C*. *B*, growth of cells ± dox-induced FLAG-SLC3A2 WTseq and variants ± 10 *μ*M fluvastatin for 48 h. *C* and *D*, cells were cultured in normal and low Glucose/Gln (10 mM/1 mM) DMEM medium with for 24 h, followed by 16 h of H_2_O_2_. Alamar Blue was added to wells for 90 min which measures reducing-potential (NAD(P)H) in viable cells, and the signal was normalized to viable cell numbers by DAPI staining and InCell imaging. The early rise in Alamar Blue signal indicates an induced resistance (IR) to low levels of H_2_O_2_ measured as area under the curve (AUC). *E* and *F*, in high nutrient conditions, cells pre-treated ± dox for 2 days to induce expression of FLAG-WTseq and variants, and in the last 24 h, without and with fluvastatin followed, by H_2_O_2_ for 16 h and reducing-potential (NAD(P)H) per viable cell measured with Alamar Blue. *G*, AP-MS/MS analysis of pulldowns from dox-induced FLAG-SLC3A2 WTseq cells (72 h) without and with 10 μM fluvastatin or 250 ng/ml swainsonine (*green*) for the final 24 h. Data is the mean ± SD of three experiments. N-glycosylated proteins are in *blue*.

Glucose starvation depletes lipid-linked oligosaccharide (Glc_3_Man_9_GlcNAc_2_-pp-dolichol), the donor substrate for OST, leading to ER stress and the unfolded protein response (7). Statins inhibit HMG-CoA reductase, thereby also lipid-linked oligosaccharide biosynthesis (77), providing an alternate means of reducing N-glycosylation independent of starvation (78). We previously reported that fluvastatin treatment of HeLa cells selectively reduces the activity of oligosaccharyltransferase at N365 and N381, while N-glycosylation at N424 was unchanged (5) (Fig. 9*A* and S9*C*). Thus, fluvastatin treatment is expected to interact with the SLC3A2 single-sites loss variants to reduce N-glycosylation and further impair SLC3A2 function.

The induced response to H_2_O_2_ was greater in WT cells than SLC3A2 KO cells in high nutrient conditions, and fluvastatin treatment reduced the response in both cell lines proportionately (Fig. 9*C*). Low nutrient conditions suppressed the response and fluvastatin had no additional effect, confirming that glucose/Gln flux is required to oppose ox-stress, and likely underpins the action of fluvastatin (Fig. 9*D*). Indeed, both SLC3A2 KO cells and fluvastatin-treated WT cells displayed imbalances in glycolysis, HBP, TCA cycle, and purine pathways, which were intensified in drug-treated KO cells (Fig. S10). SLC3A2 KO cells displayed reduced levels of cystathionine, Cys, and GSH consistent with ox-stress. Finally, dox-induced FLAG-SLC3A2 WTseq and the sites variants were equally effective at rescuing the induced-response to H_2_O_2_, whereas, with fluvastatin treatment, rescue was most impaired in order N381D > N365D > WTseq, which is proportional to SLC3A2 affinities for Gal3 as suggested by data in Figures 2 and 3. This is consistent with the relative impact of each site and cooperativity between N-glycans in retention by the Gal3 lattice (14, 58), which is revealed here by reducing N-glycosylation further in the single site mutants (Fig. 9, *E* and *F*).

In AP-MS experiments, fluvastatin treatment of cells expressing dox-induced FLAG-SLC3A2 WTseq increased association with ER protein chaperones (CANX, HSPB1, TMX3, SEC61A1, SEC61A2, HSPA5, HSP90AB1, HSP90AA1, HSPA8) (Fig. 9*G* and Table S4). These interactors were also increased with the A273N gain-of-site variant and indicate ER stress (Fig. 5*A*). CD44 was one of the most enhanced SLC3A2 interactors, ranking fourth in fluvastatin-treated cells, and is known to bind SLC3A2∗SLC7A11 under ox-stress conditions, thereby promoting surface expression, import of cystine and stress mitigation (75, 76). Although N381 and N365 sites in SLC3A2 are hyper-sensitive to statin treatment (5), CD44v has eight additional N-glycans that potentially support Gal3 binding and recruitment of SLC7A11 and AA/Na^+^ symporters as required for GSH synthesis (Gln, Glu, Cys, and Ser). Indeed, five of the enhanced SLC3A2 interactors in fluvastatin-treated cells (SLC7A11, SLC1A4, SLC38A1, SLC1A5, and SLC38A2) were also identified as depleted with N365D and N381D variants in non-stressed conditions (Figs. 9*E* and 5*B*). In replete conditions, SLC3A2 interactions require N-glycans at N365D and N381D (Fig. 5*A*), whereas reducing N-glycosylation of these sites and others with fluvastatin promotes the recruitment of CD44 along with transporters needed in the response to stress. Interestingly, SW treatment of FLAG-SLC3A2 WTseq rescued cells but had little effect on protein interactions (Fig. 9*E*), perhaps reflecting SW’s lack of effect on BCAA/Gln balance (Fig. 6, *A* and *B*). The SLC7A5 exchanger activity in HeLa cells is >2 fold that required for cell growth (Figs. S2 and 6*C*), a costly excess capacity to maintain AA balance, and presumably critical to survival under extreme conditions.

## Discussion

The recent evolution of the N-glycan positions in mammalian SLC3A2 suggests these modifications are a key feature of the adaptor function. The four NXS/T sites on human SLC3A2 display different N-glycan structures, indicating a sensitivity of *medial* and *trans-* Golgi remodeling enzymes to the microenvironment of each site. The primate-derived site at N381 and the conserved site at N424 are furthest from the membrane and have predominantly tetra-antennary N-glycans, the higher affinity ligands for Gal3 binding. Mutational analysis indicated that N-glycans at N381 and the conserved N365 site promote galectin binding, surface residency as well as endocytosis by GL-Lect (10, 12, 14, 21). Restoring a lost site at A273N enhanced branching and poly-LacNAc on the neighboring N-glycan at N365, while restoring D355N suppressed poly-LacNAc at N365. The even bias observed for poly-LacNAc units at N424 and N365 in SLC3A2 may occur with serial exposure to cell surface sialidases and *trans*-Golgi glycosyltransferases driven by GL-Lect recycling endosomes (59, 79). Supplementing cultures with GlcNAc enhanced N-glycan branching and poly-LacNAc content at N365, the site positioned to mediate endocytosis by the GL-Lect induced clathrin-independent carriers (23, 24, 25). Selection against sites at A273 and D355 leading to primates has removed their influence on N-glycan processing at N365 while retaining the capacity for regulation by HBP. SLC3A2 interacts with other glycoprotein adaptors including CD44, BSG and ATP1B-1,-3 suggesting their extracellular folds also provide the necessary spatial context for N-glycan-mediated regulation by galectins.

Membrane-anchored glycoconjugates range in size from glycolipids to the larger receptor and proteoglycans, creating vertical zones as described for β1-integrin clustering and tension with substratum (80), where the Gal3 lattice facilitates focal adhesion dynamics and cell motility (13, 22, 24). SLC3A2 N-glycans at N381 and N424 project above the membrane-embedded AA exchangers reaching >100 Å, while N365 is closer to the membrane (Figs. 1*C* and 3*G*). The SLC1A SLC38A families of AA/Na^+^ symporters have one to three conserved N-glycosylation sites in the third extracellular peptide loop where galectin cross-linking with SLC3A2 is possible. Indeed, mutation of N-glycosylation sites in SLC1A5 does not inhibit folding, transit, or transport activity but does reduce residency-time at the cell surface (81), presumably due to reduced affinity for the galectin lattice as reported for SLC7A3 and SLC2A2 (15, 82).

The SLC7A5 exchanger is fully active with or without SLC3A2 in liposomes (71), but in cells where membranes are dynamic, our results indicate that SLC3A2 N-glycans regulate Gal3-dependent endocytosis and clustering that enhances AA exchange between SLC3A2∗SLC7A5 and Na^+^/AA symporters. Nicklin *et al.* (32) have shown previously, a functional dependency between SLC7A5 and the AA/Na^+^ symporter SLC1A5. It was therefore reasonable to hypothesis that the efficiency of EAA exchange and Gln recovery by AA/Na^+^ symporters increase exponentially as a function of their proximity (Fick’s laws of diffusion). Our AP-MS experiments with site variants revealed physical interactions dependent, at least in part, on N-glycans. Cell-based assays with inhibitors of N-glycan branching (SW and Cast) are consistent with a dependency on N-glycan-mediated clustering of SLC3A2∗SLC7A5 and AA/Na^+^ symporters by the Gal3 lattice. HPB was activated 2 to 3 fold in SLC3A2 KO cells (Fig. S8), suggesting a feedback response to low intracellular EAA, directed at increasing Gal3 lattice avidity and thereby exchanger-symporter activity.

In WT HeLa cells, global inhibition of N-glycan branching with SW treatment reduced AA levels while maintaining their proportions, suggesting a weaker surface retention and/or clustering of SLC3A2∗SLC7A5 and Na^+^/AA symporters by Gal3 (Figs. 6 and S8). SW had no additional effect on the sever imbalance of BCAA, Gln, and Glu in SLC3A2 KO cells. Interestingly, SW is an alkaloid first identified more than 50 years ago in *Astragalus* weed as a hazard to grazing livestock, that causes a slowly progressing neurological disorder and reduces the efficiency of feed utilization, resulting in emaciation (83). Our results suggest that reduced N-glycan branching on transporters and adaptors may impair glutaminergic activity in the brain causing confusion (84), and reduced AA absorption from the diet (85). Interestingly, elevated Na^+^ upon imported of Glu by the excitatory AA/Na^+^ symporter (SLC1A2), induces its clustering and endocytosis (86). Inhibition of the ATPase Na^+^/K^+^ exchanger reduces N-glycan branching and growth in cancer cells (87), perhaps also by Na^+^-stimulated endocytosis of AA/Na^+^ symporters that supply Gln to HBP.

SLC3A2 interactions by AP-MS revealed oligosaccharyltransferase subunits (STT3A, DDOST, RPN1, RPN2), and the channel-forming translocon (SEC61A2, SEC61A1, SEC61B), not unexpected with transit in the secretory pathway, but perhaps suggesting an additional role as a chaperone to transports. Fluvastatin treatment shifted SLC3A2 interactions toward CD44, SLC7A11, and AA/Na^+^ symporters that are known to support GSH synthesis and oppose ox-stress (75, 76). SLC3A2 and N-glycan occupancy at N381 and N365 were required for the inducible response to low doses of H_2_O_2_, which was suppressed by pre-treatment with fluvastatin. Indeed, fluvastatin induces stress (78), and may reduce N-glycosylation at sensitive sites in other stress-related glycoproteins, notably c-AMP-dependent transcription factor (ATF6), cleavage-activating protein (SCAP) and activating endopeptidases (MBTPS1) (88, 89, 90, 91). Statins also inhibit mevalonate flux to CoQ10 biosynthesis, thereby the antioxidant ubiquinol, which along with SLC3A2∗SLC7A11 and GSH synthesis oppose lipid peroxidation and ferroptosis (92).

The evolution of Neoaves has taken the unusual path of reducing the genome size (base pairs) and purging 20 to 25% of vertebrate genes, including *SLC3A2, SLC7A5, -7, -8, -10*, and *SLC1A4, SLC1A5*, as well as *SLC43A1* and *SLC43A2*; removing this module of EAA/Gln exchangers and AA/Na^+^ symporters (Ng, Pawling and Dennis accompanying manuscript (47)). In mammals, the export of Gln with the import of EAA requires the recovery of Gln by AA/Na^+^ symporter at the cost of maintaining the Na^+^/K^+^ gradient by the ATPase exchangers. Duplication of *SLC16A* genes in Neoaves, encoding bidirectional proton-linked monocarboxylate transporters, suggest a shift from EAA exchangers to exchange of deaminated form of BCAA (ketoacid). The selection pressures leading to flight in Neoaves appear to have improved bioenergetics by reducing gene paralogues that encode burdensome cycles while duplicating genes that enhance antioxidant capacity.

In summary, SLC3A2 displays site-specific Golgi remodeling of N-glycans that regulate the opposing forces of GL-Lect-driven endocytosis and surface retention by the Gal3 lattice. Our data suggests a model where proximity mediated by the Gal3 lattice reduces the distance of diffusion between SLC3A2∗SLC7A5 exchanger and AA/Na^+^ symporters, thereby balancing AA in real time (seconds). In conditions that reduce lipid-linked oligosaccharide in ER, specific NXS/T sites are under-glycosylated by oligosaccharyltransferase and the SLC3A2 interactome shifts in composition to oppose ox-stress.

## Experimental procedures

### Materials

Antibodies to FLAG-M2 and tubulin were purchased from Sigma-Aldrich. Metabolite standards and reagents were obtained from Sigma-Aldrich with a minimal purity of 98%. ^13^C- uniformly labeled glucose and glutamine were purchased from Cambridge Isotope Laboratories, Inc. All organic solvents and water used in the sample and LC–MS mobile phase preparation were HPLC grade and obtained from Fisher Scientific. N-Glycosidase F (PNGase F, EC 3.5.1.52, recombinant cloned from *Flavobacterium* meningosepticum and expressed in *Escherichia coli*) was purchased from Roche Diagnostics. Antibodies for Western blots are rabbit anti-phospho-AMPK (T172) and AMPK with Cell Signaling #2531S and #2532S, respectively; rabbit anti-phospho-eIF2a Cell Signaling #9712S; rabbit anti-SLC3A2 (Santa Cruz Biotech. #361-375), CD98 (C-20) and sc-7095 (Santa Cruz Biotech). Anti-human CD98 antibody, clone MEM-108, Biolegend #315602. Anti-EEA-1 antibody, BD Bioscience (#610456). Fluvastatin was purchased from US Biologicals (F5277-76). CRISPR/CAS9 Reagents: FastDigest BbsI (#FD1014) from Thermo Scientific; T7 DNA ligase (#M0318S), T4 PNK (#M0201S), ATP (#P0756S) from NEB; Plasmid-Safe ATP-Dependent DNase (#E3101K) from Epicentre; PolyJet reagent from SignaGen Laboratories; CRISPR-CAS9 vector pX459. Lactose, Sigma (#L3750), and Galectin-3 inhibitor compound from Galecto, Inc (#GB0149-03), Home-produced Gal3-488 recombinant protein.

### HeLa cells

HeLa Flp-In T-REx cells were a kind gift from Dr Stephen Taylor (University of Manchester). HeLa Flp-In T-REx cell lines were cultured at 37 °C and 5% CO2 in a humidified atmosphere in high glucose DMEM (Wisent) supplemented with 10% fetal bovine serum (FBS), 2 mM Gln, penicillin/streptomycin, unless indicated otherwise. For HeLa cells with insertions of tetracycline-inducible FLAG-SLC3A2 WTseq and variants at the recombination site, expression was induced with the addition of 0.2 μg/ml doxycycline for 48 to 72 h. This was followed by the addition of 0, 5, or 10 μM fluvastatin as indicated, and cells were cultured for a further 48 h.

### CRISPR/Cas9 mutation of SLC3A2

Human SLC3A2 is a type II transmembrane glycoprotein with amino acids 1 to 184 cytoplasmic, 185 to 205 transmembrane, and 206 to 630 extracellular. SLC3A2 has four isoforms; P08195-1 with the canonical sequence of 630 amino acids; P08195-2 is missing the first 102 residues; P08195-3 is missing 38 to 99; and P08195-4 has an insertion of 32 amino acids after position 98. Two guide RNA (sgRNA) sequences were made (https://zlab.bio/guide-design-resources) to target exon four and the flanking intron for the removal of 110 bp from the SLC3A2 gene. sgRNA#1: CAGATTCAACCGGAGGTACC, and sgRNA#2: CCGCGTTGTCGCGAGCTAC. The two sgRNA sequences were separately cloned into the CRISPR-CAS9 vector px459 (93). HeLa Flp-In T-REx cells were transfected with equal amounts of each CRISPR-CAS9 px459 (sgRNA1and sgRNA2) plasmid using Lipofectamine 2000 according to manufacturer’s instructions. Following transfection, cells were cultured in DMEM (high glucose, 10% FBS) containing 1 μg/ml puromycin.

Colonies were expanded and analyzed by PCR and Western blotting. Genomic DNA was extracted using the Genomic DNA Mini Kit (Geneaid). PCR products were then analyzed by agarose gel electrophoresis. Deletions were confirmed by PCR using Fwd: AGGAGGTGGAGCTGAATGAGT Rev: CGAGACTCAGAGGAGCTGATGT primers flanking the expected deletion:707 bp without deletion, or 117 bp with deletion. PCR deletion products were sequenced. Cell lysates in Tris pH 7.5 50 mM and 2% SDS were applied to SDS-PAGE followed by Western blotting using anti-SLC3A2 (Santa Cruz, 1:5000) and anti-ϒ-tubulin antibodies (1:10,000). The transfer membranes were then probed with HRP-conjugated secondary antibodies.

### Real-time reverse transcriptase quantitative polymerase chain reaction (RT-qPCR)

RNA from cell pellets was extracted using Qiagen RNeasy kit, quantified using Nanodrop, and reverse transcribed with Superscript II. PCR was performed using the ABI Prism 7900HT Sequence Detection System (Applied Biosystems). The amplification mixture contained 1 μl cDNA and 10 μl SYBR Green PCR Master Mix (4364344; Invitrogen). Each reaction was performed in triplicate. Quantification data generated by PCR software SDS2.2.2 (Applied Biosystems) was analyzed using the ΔΔCt analysis method against GAPDH expression (94). The primers used were as follows.

**Table:** 

Target gene	Sequences	
CHOP	Forward	5′-GGAGCATCAGTCCCCCACTT
	Reverse	5′-TGTGGGATTGAGGGTCACATC
GADD34	Forward	5′-CCCAGAAACCCCTACTCATGATC
	Reverse	5′-GCCCAGACAGCCAGGAAAT
BiP/GRP78	Forward	5′-TGACATTGAAGACTTCAAAGCT
	Reverse	5′-CTGCTGTATCCTCTTCACCAGT
XBP1 (spliced)	Forward	5′-CGCTTGGGGATGGATGCCCTG
	Reverse	5′-CCTGCACCTGCTGCGGACT
XBP1 (unspliced)	Forward	5′-AGTCCGCAGCACTCAGACTACG
	Reverse	5′-TGGCAGGCTCTGGGGAAGGG
RPL13A	Forward	5′-CTTCTCGGCCTGTTTCCGTAG
	Reverse	5′-CGAGGTTGGCTGGAAGTACC
Mt-Mitochondria	Forward	5′-CACTTTCCACACAGACATCA
	Reverse	5′-TGGTTAGGCTGGTGTTAGGG
Beta2 Microglobulin	Forward	5′-TGTTCCTGCTGGGTAGCTCT
	Reverse	5′-CCTCCATGATGCTGCTTACA
GAPDH	Forward	5′-AAGGTGAAGGTCGGAGTCAAC
	Reverse	5′-GGGGTCATTGATGGCAACAATA

### Re-expression of SLC2A3 consensus and glycosylation site variants

SLC3A2 has four isoforms that differ in first ∼100 amino acids at the cytosolic N-terminus, and HeLa cells express the multiple isoforms. The N-terminal sequence is dispensable for interactions with SLC7A5 as revealed by X-ray crystallography and cryo-electron microscopy (50). To limit experimental variation in N-glycan profiling and AP-MS analysis, we made the NXS/T(X≠ P) site variants in one FLAG-tagged isoform in the HeLa Flp-In T-REx SLC3A2 KO cells cultured under standard high glucose culture conditions. Human SLC3A2 cDNA encoding amino acids 66 to 630 was FLAG-tagged at the N-terminus and cloned into the pcDNA5/FRT/TO expression vector. N-glycosylation site mutations were introduced by a combination of fusion PCR and restriction enzyme cloning using the NEB Q5 Site-Directed Mutagenesis Kit. All mutations (site deletion at N365D and N381D and site additions at A273N, D355N, D492N) were confirmed by DNA sequencing of the full encoding sequence. Each construct was cloned into the pcDNA5/FRT/TO plasmid and integrated into the genome of the HeLa Flp-In T-REx SLC3A2 KO cells described above, at a pre-integrated FRT recombination site, by co-transfection with Flp recombinase encoding POG44 plasmid, using PolyJet reagent (SignaGen Laboratories) and DMEM media without FBS. Following selection in 200 μg/ml of hygromycin, resistant clones were tested for inducible FLAG-SLC3A2 expression following 48 h of culture with 0.2 μg/ml doxycycline (dox). Cell lysates were analyzed by Western-blotting using polyclonal anti-CD98 (C-20).

### Metabolite profiling by LC-MS/MS (metabolomics)

Cells were seeded in 6-well plates with six technical replicates per experimental condition and cultured for 24 h, followed by two quick washes of the wells with warm PBS (∼37 °C), then the plates were flash frozen in liquid nitrogen (31). The metabolites were immediately extracted by adding 1 ml of extraction solution (40% acetonitrile, 40% methanol, and 20% water) and then the cells were scraped and collected in 1.5 ml vials. The mixture was shaken for 1 h at 4 °C and 1400 rpm in a Thermomixer (Eppendorfy). The samples were spun down at 14,000 rpm, for 10 min at 4 °C (Eppendorf), and supernatants were transferred into fresh tubes to be evaporated to dryness in a CentreVap concentrator at 40 °C (Labconco). The dry extract samples were stored at −80 °C until LC-MS/MS analysis. Following reconstitution in 100 μl of water containing internal standards (500 μg/ml and 300 μg/ml of D7-Glucose and ^13^C9^15^N-Tyrosine, respectively), samples were injected by auto-sampler (Dionex CorporationA) onto an HPLC column in line with a triple-quadrupole mass spectrometer (AB Sciex 5500Qtrap). Metabolites were separated through a guard column (Inertsil ODS-3, 4 mm internal diameter × 10 mm length, 3 μM particle size) and analytical column (Inertsil ODS-3, 4.6 mm internal diameter, 150 mm length, and 3-μM particle size; GL Sciences, Japan) for both polarity modes. In positive mode analysis, the organic portion (acetonitrile) of the mobile phase (0.1% acetic acid) ramped from 5% to 90% in 16 min, held for 1 min at 90%, then returned within 1 min to 5% acetonitrile in mobile phase for column regeneration. In negative mode, the acetonitrile composition ramped from 5 to 90% in 10 min, held for 1 min at 90%, then the gradient ramped back over 3 min to 5% acetonitrile in the mobile phase (0.1% tributylamine, 0.03% acetic acid, 10% methanol), to regenerate the column for the next run. The total runtime for each sample in both modes was 20 min and the samples were maintained at 4 °C in the auto-sampler, and the injection volume was 10 μl. An automated washing procedure was developed before and after each sample to avoid sample carryover. The mass spectrometric data acquisition time for each run was 20 min, and the dwell time for each MRM channel was 10 milliseconds. Settings for negative and positive modes with electrospray ionization (ESI) were optimized. MultiQuant software (AB Sciex, Version 2.1) was used for peak analysis of ∼120 targeted metabolites, with standards run consecutively. Peaks representing targeted masses and LC retention times were confirmed manually. Common mass spectrometric parameters: GS1 and GS2 were 50 psi; CUR was 20 psi, and CAD was three and seven for positive and negative modes, respectively, and source temperature (TEM) was 400 °C. The signal was normalized to internal standard and cell number. MetaboAnalyst was used for preliminary statistical analysis of the LC-MS/MS data (https://www.metaboanalyst.ca/MetaboAnalyst/ModuleView.xhtml) (95). The LC-MS/MS system does not resolve hexose and hexosamine isomers including glucose/galactose and GlcNAc/GalNAc. To monitor trends in metabolic pathways, we refer to these isomers in their glucose (Glc) forms.

### Cell migration by scratch-wound assay

Cells were seeded into 6-well plates and serum starved for 24 h, as described. A P10 pipette tip was used to create a linear wound, and healing by cell migration was monitored for 24 h using time-lapse microscopy *via* an inverted microscope (DMIRE2; Leica Microsystems) with a motorized stage and live-cell apparatus (37 °C humidified chamber with 5% CO2; Applied Scientific Instrumentation). Images were captured every 15 min using an ORCA Hamamatsu CCD camera (Hamamatsu Corporation) with a × 10 lens. Image analysis was carried out by Velocity 3D Image Analysis Software (Improvision) by measuring the total wound area in three fields per condition, at t = 0, 4, 6, 8,12, and 24 h. Measurements at each location were averaged to yield a mean wound area. The residual wound area was expressed as a percent of the original wound.

### Cell growth

Cell lines were plated in 96-well Nunc 165,305 Optical-Bottom plates at 1000 cells/well and growth was measured daily as a fraction of well confluence using the Celigo Cell Cytometer’s label-free bright field imaging. Scans were repeated daily until confluence reached approximately 100% and data was graphed as a fraction of confluence with time. Cell growth was also analyzed using Alamar Blue reagent (Invitrogen), and plate reader (Gemini Fluorescence from Molecular Devices) at fluorescent excitation 590 nm and emission at 544 nm.

The induced response to ox-stress was also measured with Alamar Blue, which reacts with reducing-potential (*i.e.*, NAD(P)H). HeLa cells with insertions of tetracycline-inducible FLAG-SLC3A2 at the recombination site were induced by the addition of 0.2 μg/ml doxycycline for 48 h, after which they were plated at 7000 cells per well in 96 well optical plates in rows of 12 replicates. Control wells of non-Tet-induced cells were usually included in each assay. After 24 h in the 96 well plates, freshly diluted H_2_O_2_ was titrated at 0 to 600 μM across the 12 replicate wells and incubated for 16 h. Alamar Blue and Hoescht were added to each well and the Alamar Blue signal was read as above after 1 and 4 h and normalized to cell number by Hoescht staining and counting of nuclei by InCell imaging.

### Export and recovery of amino acid from the medium in restricted conditions

Media samples for metabolomics analysis were prepared from 1.2 × 10^6^ cells plated in 6-well plates and allowed to adhere in complete media for 12 h. Cells were briefly rinsed with PBS (with Ca2+/Mg2+) and then incubated with AA-free DMEM/F12 media (US Biological) for 3 h. Fresh amino acid-free DMEM/F12 supplemented as indicated with 3% BSA alone or + 2 mM Gln and then media aliquots were taken at the indicated time points. Aliquots were centrifuged at 16,100 g for 5 min, transferred to fresh tubes, and stored at −80 °C until further processing. For targeted metabolomics by LC-MS/MS, 10 μl media aliquots (thawed just until melted) were extracted with 500 μl ice-cold extraction solvent (40% acetonitrile:40% methanol:20% H_2_O) and vortexed for 1 min, then shaken in an Eppendorf shaker (Thermomixer R) at 1400 rpm, 4 °C for 20 min and centrifuged at 4 °C for 15 min at 16,100 g. Supernatants were transferred to a clean Eppendorf tube, evaporated to dryness in a CentreVap concentrator at 40 °C stored at −80 °C, then resuspended in 180 μl of water containing the Internal Standards and analyzed by LC-MS/MS as described above.

### Antibody binding and uptake experiment

Cells were shifted on ice for 10 min on ice (4 °C) and washed 3 times with ice-cold DMEM. 10 μg/ml of anti-SLC3A2 (MEM-108 clone) prepared in the same medium was added to the cells for 30 min incubation. Excess of antibody was removed by three successive washes with ice-cold DMEM medium. Cells are then PFA-4% fixed and immuno-stained against the bound antibody. In the case of co-binding assay (*i.e.*, anti-SLC3A2 and Gal3), 5 μg/ml of recombinant Gal3-488 was primarily bound on the cooled cells for 30 min prior to switching to the antibody binding step. Three ice-cold DMEM washes are required between Gal3 binding and antibody binding steps.

Continuous antibody uptake was performed by incubating cells with 10 μg/ml of anti-SLC3A2 (MEM-108 clone) for 10 min at 37 °C. Cells were then shifted to 4 °C on ice, 3 times washed with ice-cold-PBS, then PFA-4% fixed, saponin-permeabilized, and immuno-stained against the uptaken antibody. For co-uptake experiment, Gal3 and anti-SLC3A2 were sequentially bound on pre-cooled cells, as described above, and then shifted to 37 °C for 10 min internalization.

### SLC3A2/Gal3 interaction

The experiment was performed as described earlier for the co-binding experiment except that here the cells were lysed. The PNS-cleared lysate was then overnight incubated at 4 °C with protein G-sepharose beads for SLC3A2 immuno-precipitation (IP). Samples were denatured and eluted from the beads for SDS-PAGE analysis. Co-pulled down Gal3 was further quantified.

### Gal3 inhibition using GB0149-03 or lactose

For acute treatment, cells were pre-treated with 10 μM GB0149-03 compound or 150 mM lactose for 5 to 10 min at 37 °C. The inhibitors were washed out during the following antibody uptake experiment.

For severe treatment, cells were pre-incubated with 10 μM GB0149-03 compound or 150 mM lactose for 30 min and 60 min at 37 °C. In contrast with the acute treatment, here the inhibitors were kept during the internalization step.

### Extract of endogenous SLC3A2 glycopeptides from membrane proteins

HeLa cells (∼2 × 10^6^) were suspended in 1 ml of homogenization buffer (0.25 M sucrose, 50 mM HEPES pH 7.5, 5 mM NaF, 5 mM EDTA, 2 mM DTT, cOmplete protease inhibitor), and lysed using a probe sonicator. Homogenate was cleared at 2000*g* for 20 min at 4 °C, then ultracentrifuged at 115,000*g* for 70 min at 4 °C. The pellet was vigorously suspended in 650 μl Tris buffer (0.8% Triton X-114, 50 mM Tris pH 7.5, 0.1 mM NaCl, 5 mM EDTA, 5 mM NaF, 2 mM DTT, cOmplete protease inhibitor). The homogenate was chilled on ice for 10 min, incubated at 37 °C for 20 min, then phase partitioned at 1950*g* for 2 min at room temperature. The upper phase was discarded. Membrane proteins in the lower phase were precipitated with 1 ml acetone at −20 °C overnight. After centrifugation at 1950*g* for 2 min, the precipitated membrane proteins were stored at −25 °C.

### SDS gel separation of endogenous SLC3A2 and in-gel digest

SDS-PAGE gel loading buffer (250 uL) was added to extracted membrane pellets from HeLa cells, agitated, and heated 2 min at 100 °C to denature proteins. Proteins were separated on 8% SDS-PAGE gels, stained with GelCode Blue, and bands between 80 to 90 Da (band 1), 90 Da to 110 Da (band 2), and 110 Da to 130 Da (band 3) were cut. The gel pieces were washed with 50 mM ammonium bicarbonate (ABC) and acetonitrile (ACN) 4 times, without reduction and alkylation, followed by in-gel digest with 25 ng/μl trypsin in 50 mM ABC overnight at 37 °C. Tryptic peptides were extracted from the gel pieces with 25 mM ABC and 0.5% FA, then dried by speed vac. Tryptic peptides were dissolved in 20 μl 0.5% FA and de-sialidated with sialidase and processed for glycopeptide identification and site-specific N-glycan analyses.

### Glycopeptides enrichment

HILIC microtips were prepared with 10 mg of PolyHYDROXYETHYL A (PolyLC Inc), in a bed volume of 50 μl, washed with 500 μl of ddH_2_O and equilibrated with 500 μl HILIC solvent (80% ACN, 0.1% TFA). Dried tryptic peptides were dissolved in 100 μl HILIC solvent, loaded slowly onto microtips, and spun down at 700 to 1000 rpm. Samples were loaded a second time into the microtip then washed 3 times with 1 ml HILIC solvent, and glycopeptides eluted with 500 μl of 100 mM ammonium bicarbonate. The eluted glycopeptides were speed vacuumed to dry, and analyzed by LC-MS and MS/MS.

### Glycopeptide preparation for dox-induced FLAG-SLC3A2

Dox-induced FLAG-SLC3A2 variant cell lines (∼10^7^) were lysed in 1 ml of lysis solution (1% Triton100, 20 mM Tris pH7.5, 150 mM NaCl, 1 mM EDTA and 1 mM EGTA, with freshly prepared protease inhibitors), and centrifuged at 14,000 rpm for 30 min to pellet the nuclei and insoluble material. A 15 μl aliquot was suspended in an SDS loading buffer, heated at 100 °C for 1 min, and followed by SDS-PAGE and Western blotting to confirm induced expression of FLAG-SLC3A2. Protein concentration was determined by Thermo BCA protein assay. Normalized protein 1 mg lysates were incubated with anti-FLAG M2 antibody-conjugated agarose 20 μl (bed volume, from 40 uL FLAG beads slurry (1:1)) overnight on a rotating platform at 4 °C. The beads were washed 4 times (1 ml) with TBS (50 mM Tris pH7.5, 150 mM NaCl) and 3 times with 50 mM ammonium bicarbonate (ABC, 0.8 ml) before re-suspending in 20 ml ABC (50 mM). For on-beads digest, trypsin (enzyme ∼0.5 μg) was directly added to the beads and incubated at 37 °C overnight. The next morning, 0.25 ug trypsin was added again and digested for another 2 h, then heated at 100 °C for 1 min to inactivate trypsin. Peptide was extracted with 50 μl 0.5% formic acid 3 times and dried by speed vac. Samples were dissolved with 10 μl 50 mM ABC with 0.5 μl sialidase and incubated at 37 °C overnight. Half of the trypsin digest was used for in-solution Asp-N digest (Asp-N (120 ng, 40 μg/μl), 37 °C overnight digest.) Both trypsin and trypsin plus Asp-N digest samples were injected (0.5 μl) for LC-MS/MS glycopeptide identification and glycopeptide quantification by LC-MS.

### Glycopeptide analysis by LC-MS and MS/MS

Tryptic peptides dissolved in 100 μl HILIC solvent were prepared on HILIC microtips. Samples were then applied to a nano-HPLC Chip using an Agilent 1200 series microwell-plate autosampler, interfaced with an Agilent 6550 iFunnel Q-TOF MS (Agilent Inc). The reverse-phase nano-HPLC Chip (G4240-62002) had a 40 nl enrichment column and a 75 μm × 150 mm separation column packed with 5 μm Zorbax 300SB-C18. HILIC retains hydrophilic glycopeptides. For HILIC-enriched hydrophilic glycopeptide, mobile phase began with 2% ACN. For hydrophobic glycopeptides, mobile phase began with 8% ACN. The mobile phase was 0.1% formic acid in water (v/v) as solvent A, and 0.1% formic acid in ACN (v/v) as solvent B. The flow rate at 0.3 μl/min with gradient schedule; 2% B (or 8% B) (0–0.2 min); 2 to 40% B (or 8–40%) (0.2–35 min); 40 to 80% B (35–42 min); 80% B (42–45 min) and 80 to 2% B (45–50 min). The MS system was in positive ion mode with 2 GHz Extended Dynamic Range mode: V^cap^: 1800 to 1900 V; drying gas flow 5.0 L/minute at T = 280 °C; fragmentor voltage 360 V: precursor selection 10 precursors/cycle; threshold 1000 counts abs and 0.001% rel; active exclusion after two spectrum; and release after 0.5 min to start again. Internal reference mass calibration used m/z 1221.9906 (Agilent), 445.1200 and 741.1951 (Polysiloxane). LC-MS/MS targeting specific glycopeptides was done to confirm the glycosylation site and glycan structure. For glycopeptide quantification, run in MS mode only, mass range was set to standard (3200 *m/z*).

Peptide identification was done with mass range set to narrow (Low 1700 *m/z*); auto MS/MS at 8 MS (range 350–1700 *m/z*), 3 MS/MS (range 50–1700 *m/z*) per/s; narrow isolation width (1.3 *m/z*), with collision energy determined on the fly using a slope of 3.6 and intercept of −4.8. Glycopeptide MS/MS analysis of N-glycan structures used mass range set to standard (3200 *m/z*); auto MS at 8 MS (range 700–2500 *m/z*), MS/MS (range 100–3000 *m/z*) per/s; narrow isolation width (1.3 *m/z*), with collision energy determined on the fly using a slope of 1.8 and intercept of −4.8. Targeted glycopeptide analysis was done with a mass range set to standard (3200 *m/z*); targeted MS/MS was set to different CE for inclusion of targeted glycopeptides. Data analysis by Mascot search was used to identify proteins and peptide sequences. Glycopeptides were identified by the presence of hexose and N-acetylhexosamine using Agilent Masshunter Quantitative Analysis software (B06.01) and glycan structures were predicted for extracted glycopeptides by online GlycoMod (http://web.expasy.org/glycomod/) and confirmed manually.

### Total cellular N-glycan profiling

Precipitated membrane protein (30 μg) was suspended in 60 μl of 0.25% RapiGest SF, 50 mM ammonium bicarbonate, 5 mM DTT, heated at 85 °C for 3 min, then mixed with 1 μl of PNGase F, 0.7 μl of sialidase, and 20 μl of 50 mM ammonium bicarbonate, and incubated at 42 °C for 2 h followed by 37 °C overnight. Released *N*-glycans were extracted with 4 to 5 volumes of 100% ethanol at −80 °C for 2 h. The supernatant containing released *N-*glycans was speed vacuumed to dry. Pipet tips packed with 10 mg porous graphitized carbon (PGC) for a bed volume of 50 μl were washed with 500 μl of ddH_2_O, 500 μl of 80% acetonitrile (ACN), and equilibrated with 500 μl 0.1% trifluoroacetic acid (TFA). *N-*glycan pellets were dissolved in 50 μl of 0.1% TFA and slowly loaded into the microtips at a flow rate of ∼100 μl/min, washed with 500 μl 0.1% TFA, and *N-*glycans eluted with 500 μl of elution buffer (0.05% TFA, 40% ACN). The eluted *N-*glycans were analyzed by LC-MS/MS. Total N-glycan samples were applied to a nano-HPLC Chip using an Agilent 1260 series microwell-plate autosampler and interfaced with an Agilent 6550 iFunnel Q-TOF MS (Agilent Technologies, Inc). The HPLC Chip (glycan Chip) had a 40 nl enrichment column and a 75 μm × 43 mm separation column packed with 5 μm graphitized carbon as the stationary phase. The mobile phase was 0.1% formic acid in water (v/v) as solvent A, and 0.1% formic acid in ACN (v/v) as solvent B. The flow rate at 0.3 μl/minute with gradient schedule; 5% B (0–1 min); 5 − 20% B (1–15 min); 20 to 70% B (15–16 min); 70% B (16–19 min) and 70 to 5% B (19–20 min). The MS System was operated in positive ion mode at 2 GHz Extended Dynamic Range, MS mode in low mass range (1700 m/z) with MS setting at 8 MS (range 450–1700 *m/z*).

#### Data analysis

PNGase F-released free N-glycan was identified by Agilent Masshunter Quantitative Analysis software by the presence of hexose and N-acetylhexosamine. N-glycan structures were predicted by online GlycoMod (http://web.expasy.org/glycomod/). Finally, Agilent Masshunter Quantitative Analysis software was used to quantify the extracted glycan peaks.

### SLC3A2 interactors by AP-MS

HeLa Flp-In T-REx cells with insertion of dox-inducible FLAG-SLC3A2 were seeded (2 × 10^6^ cells per 10 cm plate) in DMEM high glucose media plus 10% FBS, followed 24 h later by the addition of 0.2 μg/ml doxycycline for 48 h. Cells were pelleted and stored at −80 °C. Cells were lysed in 250 μl FLAG lysis buffer (0.5% NP40, 50 mM HEPES pH 8.0, 100 mM KCl, 1 mM EDTA, 10% glycerol with fresh protease inhibitors) as described (96). Samples were frozen and thawed once then agitated in 37 °C water bath to thaw and centrifuged at 14,000 rpm for 30 min to pellet the nuclei and insoluble material. An aliquot of cell lysate was suspended in an SDS loading buffer, heated at 100 °C for 1 min, and followed by PAGE and Western blotting with anti-SLC3A2 antibody to check the SLC3A2 expression (protein concentration was determined by Bradford analysis). For proteomics, 1 mg of protein in ∼240 μl lysate was incubated with 40 μl anti-FLAG M2 antibody-conjugated agarose slurry (1:1) for 4 h on a rotating platform at 4 °C. The beads were washed 2 times (1 ml) with FLAG lysis buffer, 2 times with FLAG Rinsing Buffer (20 mM Tris pH 8.0 and 2 mM CaCl_2_) and 2 times with 50 mM ammonium bicarbonate (ABC, 0.8 ml) before re-suspending in 20 ml ABC (50 mM) for on-beads trypsin digest.

### Data-dependent acquisition and SWATH

Samples were prepared for protein identification by DAA and quantification by SWATH using TripleTOF Mass Spectrometer as described (97). Briefly, each sample (5 μl) was directly loaded onto an equilibrated HPLC column at 400 nl/minute flow rate. The peptides were eluted from the column over a 90 min gradient generated by a NanoLC-Ultra 1D plus (Eksigent) nano-pump and analyzed on a TripleTOF 600 instrument (AB SCIEX). The gradient was delivered at 200 nl/minute starting from 2% acetonitrile with 0.1% formic acid to 35% acetonitrile with 0.1% formic acid over 90 min followed by a 15 min cleanup at 80% acetonitrile with 0.1% formic acid, and a 15 min equilibration period back to 2% acetonitrile with 0.1% formic acid, for a total of 120 min. To minimize carryover between each sample, the analytical column was washed for 3 h by running an alternating sawtooth gradient from 35% acetonitrile with 0.1% formic acid to 80% acetonitrile with 0.1% formic acid, holding each gradient concentration for 5 min. Analytical column and instrument performance were verified after each sample by loading 30 fmol bovine serum albumin (BSA) tryptic peptide standard (Michrom Bioresources) with 60 fmol a-casein tryptic digest and running a short 30 min gradient. TOF MS calibration was performed on BSA reference ions before running the next sample to adjust for mass drift and verify peak intensity. The TripleTOF 600 method was set to data dependent acquisition (DDA) mode, which consisted of one 250 ms (ms) MS1 TOF survey scan from 400 to 1300 Da followed by 20,100 ms MS2 candidate ion scans from 100 to 2000 Da in high sensitivity mode. Only ions with a charge of 2+ to 4+ that exceeded a threshold of 200 cps were selected for MS2, and former precursors were excluded for 10 s after one occurrence. Half of the sample was analyzed by DDA as above, and the other half was analyzed as described below by DDA (SWATH).

For SWATH, acquisition consisted of one 50 ms MS1 scan followed by 54 dynamic isolation windows covering the mass range of 400 to 1250 AM.u. (cycle time of 3.25 s); an overlap of 1 Da between SWATH was preselected. The collision energy for each window was set independently as defined by CE = 0.06 3 m/z + 4, where m/z is the center of each window, with a spread of 15 eV performed linearly across the accumulation time.

### Protein identification

Mass spectrometry data were stored, searched, and analyzed using the ProHits laboratory information management system (LIMS) platform (98). DDA data files were searched using Mascot against human protein database with the RefSeq database (version 57, NCBI) against a total of 72,482 human and adenovirus sequences supplemented with common contaminants from the Max Planck Institute (http://141.61.102.106:8080/share.cgi?ssid=0f2gfuB) and the Global Proteome Machine (GPM; https://www.thegpm.org/crap/index.html). Database parameters were set to search for tryptic cleavages, allowing up to two missed cleavage sites per peptide with a mass tolerance of 40 ppm for precursors with charges of +2 to +4 and a tolerance of ± 0.15 AM.u. for fragment ions. Deaminated asparagine and glutamine and oxidized methionine were allowed as variable modifications.

SWATH data file was analyzed with MSPLIT in ProHits. Library was generated in-house using DDA files searched with MS-GFDB for trypsin digest, revealing all cleavage sites with a mass tolerance of 50 ppm for parent ions (http://proteomics.ucsd.edu/Software/MSGFDB/) and set as DDA mascot search. Only oxidized methionine was allowed as a variable modification. MSPLIT-DIA parameter settings were: FDA, 0.01; fragment mass tolerance, 50 ppm; retention time window 10 min; variable SWATH window from 400 to 1250 (Table S4). After the MSPLIT analysis of SWATH data, spectral counts with three or more unique peptides detected per protein in dox-induced SLC3A2 (WTseq) and near absent in SLC3A2 KO cell samples were used to generate a preliminary list of 156 SLC3A2 interactors. Running MSPLIT-DIA prior to targeted extraction by Skyline restricted the search space by providing accurate retention times and a list of the peptides expected to be in the sample.

### Targeted data extraction from SWATH MS/MS using skyline

Skyline (http://proteome.gs.washington.edu/software/skyline) is an open-source, Windows-based software for curating and analyzing data from proteomic experiments. A primary list of 185 sequences of interest was retrieved from Uniprot and uploaded onto Skyline to create a background Proteome Database. Detailed Skyline and data processing settings are given in (99). Briefly, the spectral library generated from DDA files was uploaded in Skyline, and SWATH-MS data files were processed using the full scan MS/MS filtering at a resolving power of 30,000. Unique peptides were refined and curated for reproducible fragment ions. Peak boundaries for each selected peptide were manually supervised and when necessary, adjusted. The reproducibility and reliability of selected peptides and transitions were verified visually by looking at the ion peak-to-area ratio across the samples. We used at least three peptides per protein and at least three fragment ions per peptide for every protein. The extracted transition peak areas were exported to Excel as raw intensities and normalized to the intensity of SLC3A2 tryptic peptides. The total intensity of representative peptides for each protein was summed, then divided by the number of transitions, and the resulting average intensity was used to draw comparisons for SLC3A2 interactors (for the same gene, same parent ion, same fragment). The normalized intensity was then converted to a percentage (relative abundance), using the highest value in each experiment. The relative abundance of transitions representing the same peptides for each protein was averaged and presented as protein level and used to compare fold change for SLC3A2 interactors across all mutants and replicas. Data was normalized to the highest XIC (extracted ion chromatogram), as a reference to the maximum value across all samples. The Kruskal-Wallis test was used to compare data for WTseq and all mutations to determine significant differences, then the Dunn test was applied for pairwise comparison eliminating two that were not different. *p* ≤ 0.05.

## Data availability

Proteomics Data has been deposited as a complete submission to the MassIVE repository (https://massive.ucsd.edu/ProteoSAFe/static/massive.jsp) and assigned the accession numbers MSV000092980, MSV000092985, MSV000092988, MSV000092989.

## Supporting information

This article contains supporting information.

## Conflicts of interests

The authors declare that they have no known competing financial interests or personal relationships that could have appeared to influence the work reported in this paper.

## Supporting information

Supplemental Figs. S1–S10 and Tables S1–S6 Captions
