# Supplementary material for: SLC3A2 N-glycosylation and Golgi remodeling regulate SLC7A amino acid exchangers and stress mitigation
Source: J Biol Chem. 2023 Nov 2;299(12):105416. doi: 10.1016/j.jbc.2023.105416 (PMC10698284; doi:10.1016/j.jbc.2023.105416)
Supplement: Supplemental Figs. S1–S10 and Tables S1–S6 Captions [file mmc1.pdf]

Supporting Information

SLC3A2 N-glycosylation and Golgi remodeling regulates SLC7A amino acid exchangers and stress mitigation.

Cunjie Zhang[1], Massiullah Shafaq-Zadah[6], Judy Pawling[1], Geoffrey G. Hesketh[1], Estelle Dransart[6], Karina Pacholczyk[1], Joseph Longo[4,5], Anne-Claude Gingras[1,2], Linda Z. Penn[4,5], Ludger Johannes[6], and James W. Dennis[1,2,3,#]

**Figure S1**	Transporters and evolution of SLC3A2 N-glycosylation sites
**Figure S2**	SLC3A2 KO cell phenotype
**Figure S3**	N-glycan profiles on endogenous SLC3A2
**Figure S4**	Detailed analysis of N-glycans by MS/MS at N381 and N424
**Figure S5**	GlcNAc treatment increased branching selectively at N365
**Figure S6**	Gal3-SLC3A2 accumulation in perinuclear EEA-1 positive endosomes
**Figure S7**	Dox-induced FLAG-SLC3A2 promotes resistance to ox-stress
**Figure S8**	Golgi N-glycan processing and feedback to HBP and AA balance
**Figure S9**	BSA catabolism as the source of AAs
**Figure S10**	Metabolic profile of Fluvastatin treated WT and SLC3A2 KO cells

**Table S1**	Site-specific FLAG-SLC3A2 WTseq and variants.
**Table S2**	Site-specific N-glycan SLC3A2 endogenous
**Table S3**	Site-specific FLAG-SLC3A2 _GlcNAc-supplement
**Table S4**	SWATH Proteomics with FLAG-SLC3A2
**Table S5**	Swain Cast WT and SLC3A2 KO metabolites
**Table S6**	Total N-glycans WT and SLC3A2 KO

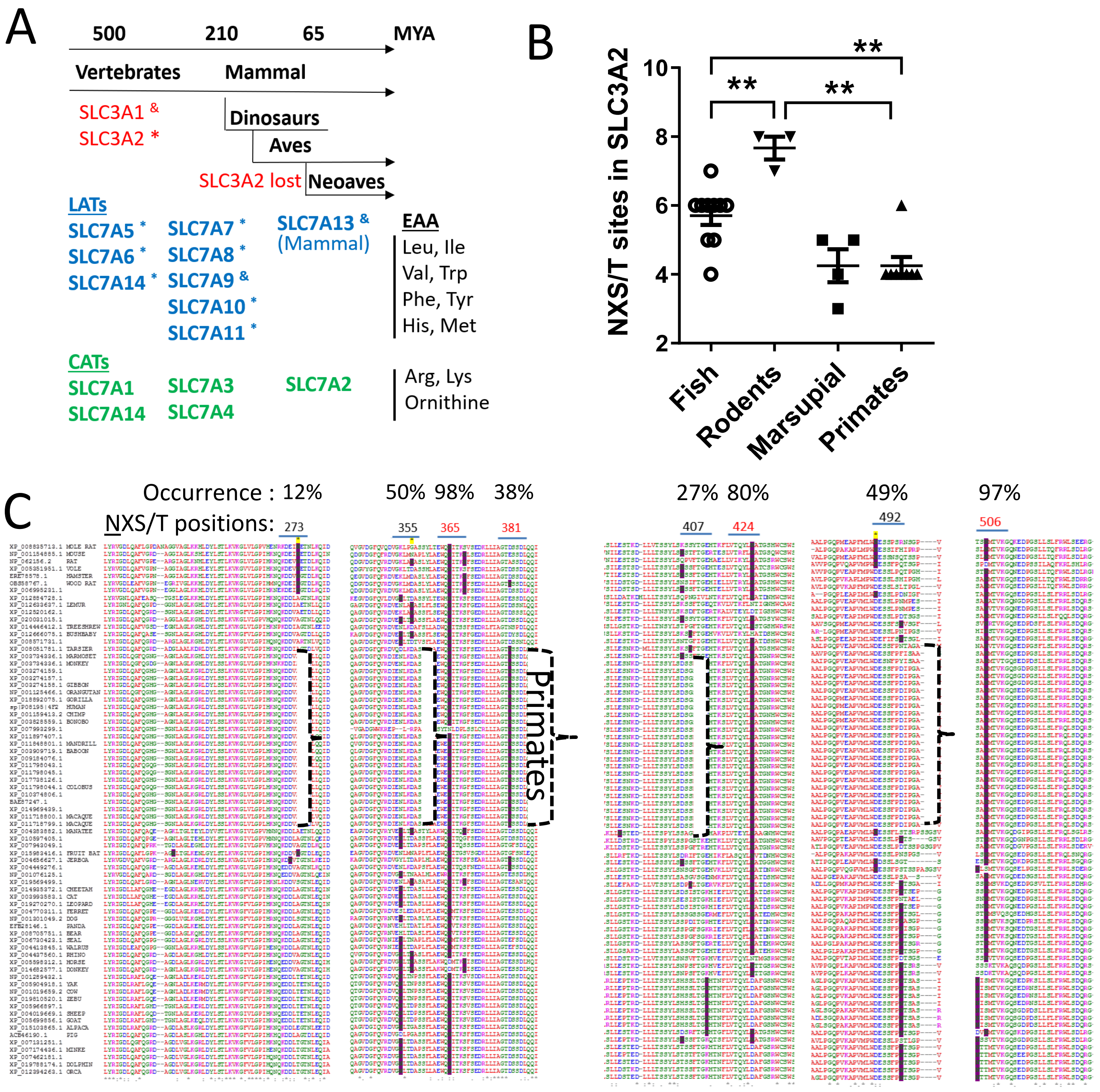

Figure S1: Transporters and evolution of SLC3A2 N-glycosylation sites (A) SLC7A family of sodium-independent transporters. The N-glycosylated adaptor SLC3A2* and SLC3A1& heterodimerize with non-glycosylated large amino acid transporters (LATs) as indicated by the symbol. The cationic amino acid transporters (CATs), SLC7A1-4 and 14 are N-glycosylated and do not heterodimerize with an adaptor. On the right: an incomplete guide to AA **s**ubstrates for LATs and CATs. **(B)** N-glycosylation site number in SLC3A2 from fish (n=10 species), rodents (n=4), marsupials (n=4) and primates (N=8) ** Dunnett's multiple comparison p <0.01 (see Fig. S1A). **(C)** Alignment of mammalian SLC3A2 sequences with the N of NXS/T(X≠P) marked (dark red fill) to reveal recent evolution of N-glycosylation sites. Variation in site position are observed in 3-6 AA regions, and four sites have been lost in the human sequence. Brackets denote the four lost ancestral NXS/T sites and the primate derived site at N381. The sites at N365, N424, N492 are more conserved.

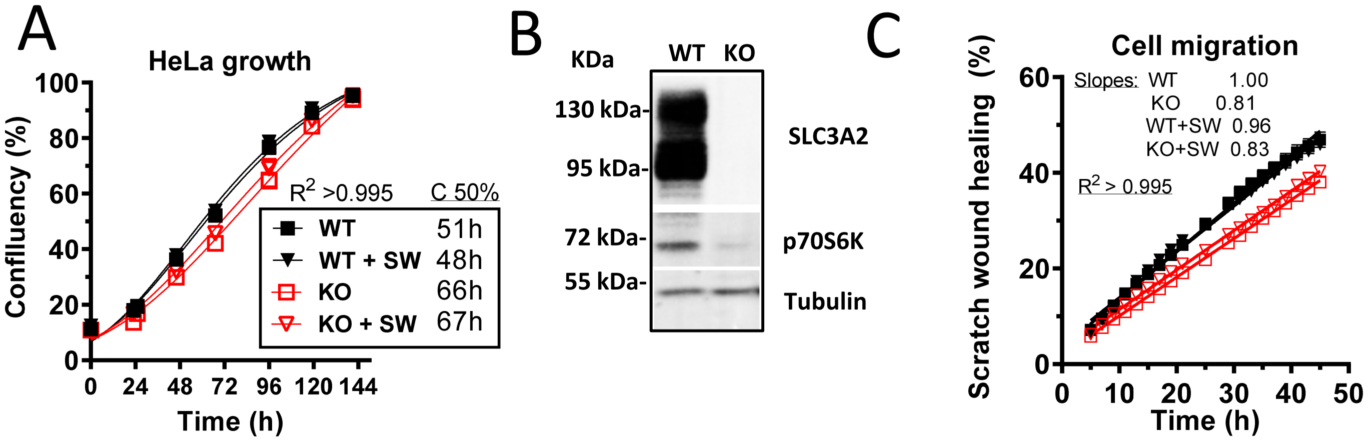

Figure S2: SLC3A2 KO cell phenotype (A) Growth of WT and SLC3A2 KO HeLa cells cultured in normal conditions of DMEM +10% FCS with and without 250nM swainsonine. **(B)** Western blot with anti-SLC3A2 antibody for lysates of WT and KO cell in log phase growth. **(C)** Cell motility in a lane-scrape wound healing assay.

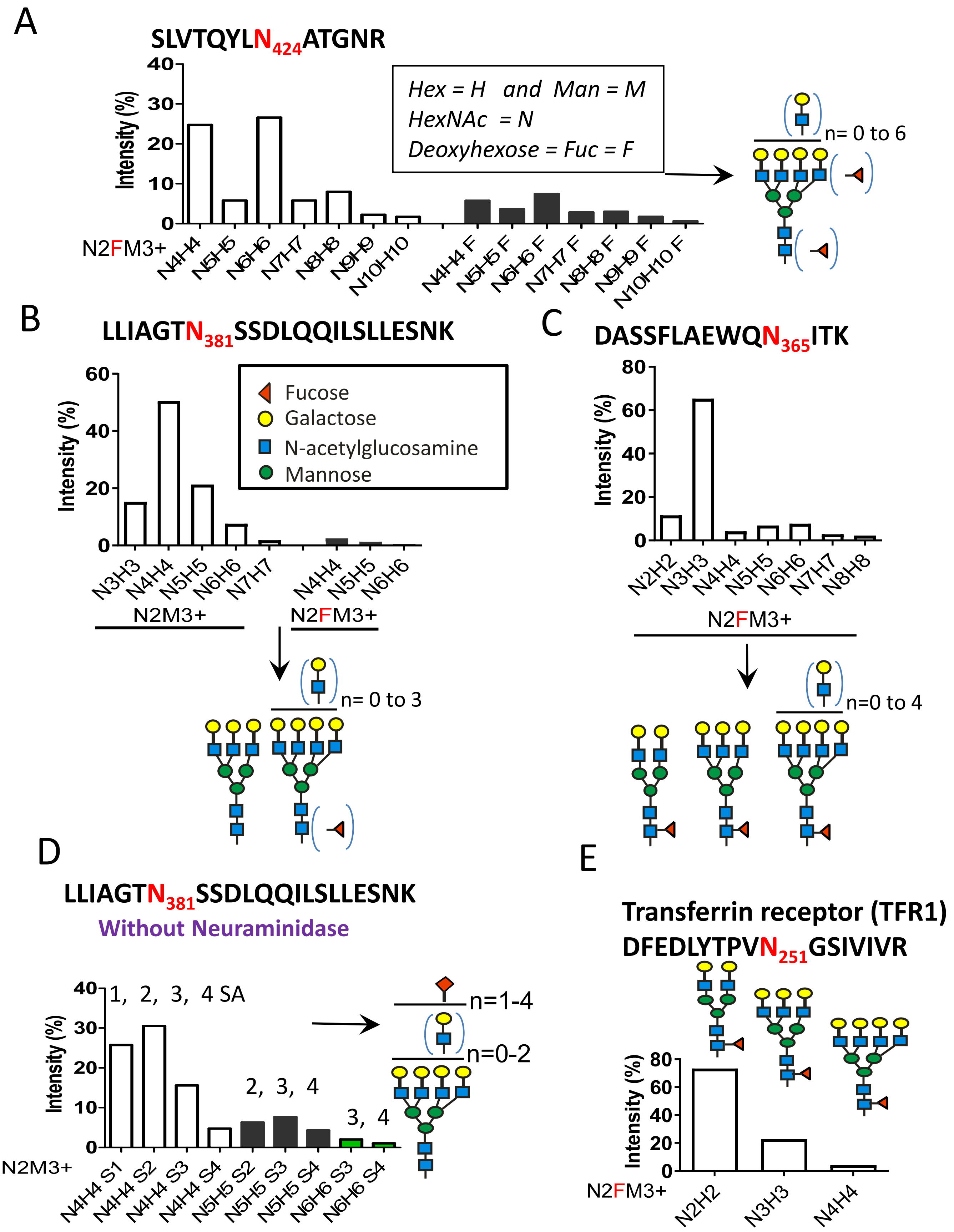

Figure S3: N-glycan profiles on endogenous SLC3A2 . (A,B,C) Glycoproteins from cell membrane preparation were separated by PAGE, and native SLC3A2 eluted from gel fractions. Tryptic peptides for three of the four sites in SLC3A2 were accessible to LC-MS/MS analysis. Sialic acid was removed with neuraminidase pre-treatment. **(D)** Analysis of the N381 glycopeptide without removing sialic acid, revealed a profile consistent with panel B. **(E)** By way of comparison, a transferrin receptor N-glycans at N251 from the same HeLa membrane preparation displayed ~75% bi-, 20% tri and <5% tetra- antennary N-glycans, proportions closer to that of the total N-glycan pool (Table S2).

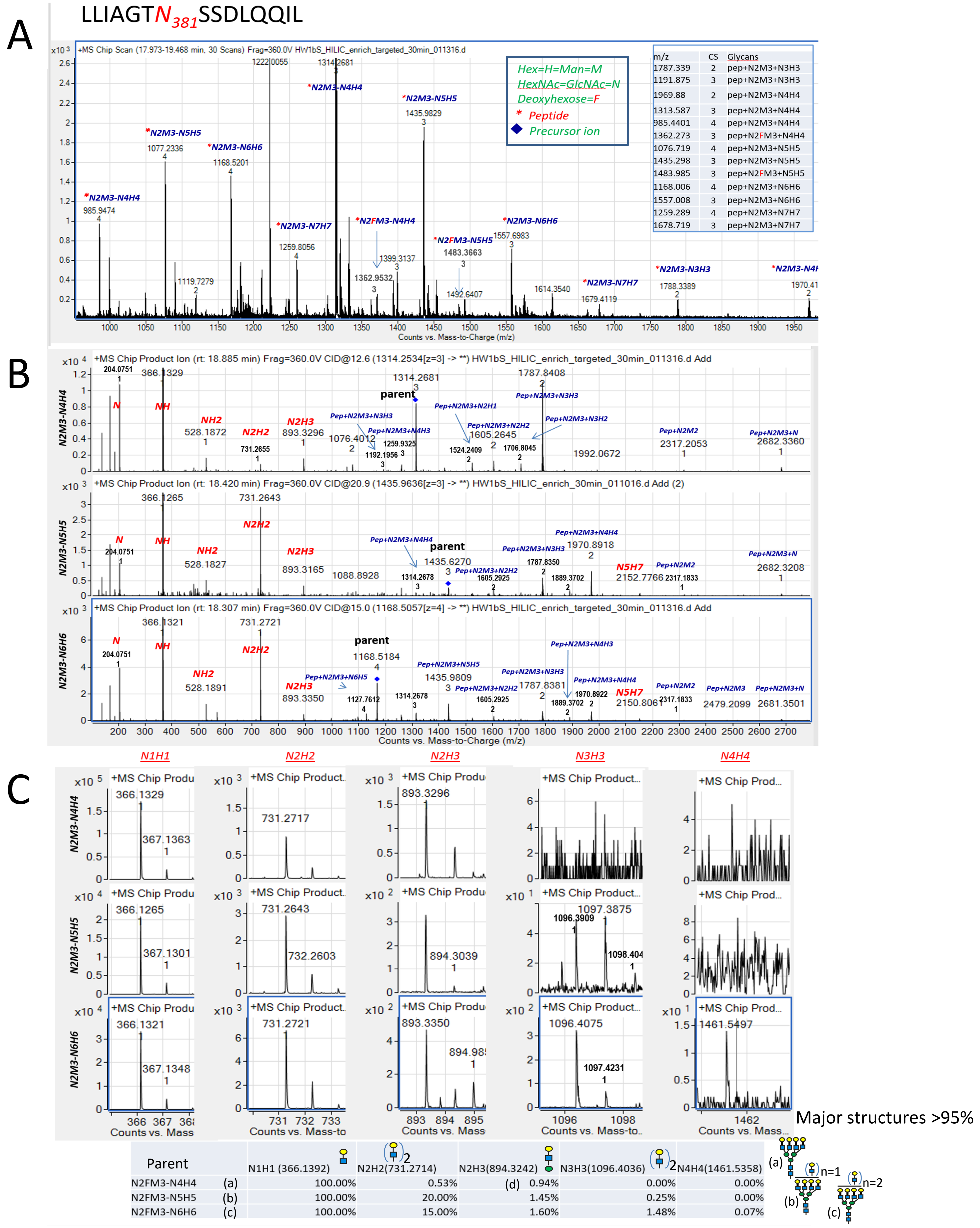

Figure S4: Detailed analysis of N-glycans by MS/MS at N381 and N424. (A,D) MS profile to identify major N-glycan species at N381. **(B,E)** Fragments identify fucose in core N-glycan and poly-LacNAc repeats. **(C,F)** Expansion of M/Z axis identifies stereoisomers of identical structures. The supplemental table S1 summarizes an interpretation of the results.

D

SLC3A2: SLVTQYLN_{424}ATGNR

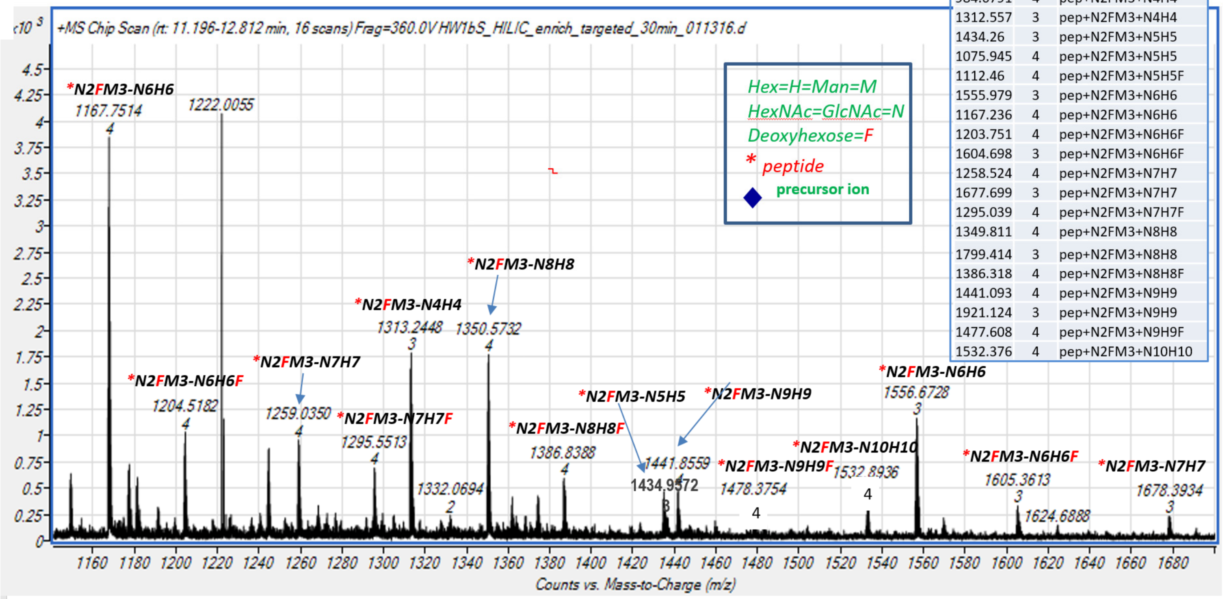

m/z	CS	Glycans
984.6791	4	pep+N2FM3+N4H4
1312.557	3	pep+N2FM3+N4H4
1434.26	3	pep+N2FM3+N5H5
1075.945	4	pep+N2FM3+N5H5
1112.46	4	pep+N2FM3+N5H5F
1555.979	3	pep+N2FM3+N6H6
1167.236	4	pep+N2FM3+N6H6
1203.751	4	pep+N2FM3+N6H6F
1604.698	3	pep+N2FM3+N6H6F
1258.524	4	pep+N2FM3+N7H7
1677.699	3	pep+N2FM3+N7H7
1295.039	4	pep+N2FM3+N7H7F
1349.811	4	pep+N2FM3+N8H8
1799.414	3	pep+N2FM3+N8H8
1386.318	4	pep+N2FM3+N8H8F
1441.093	4	pep+N2FM3+N9H9
1921.124	3	pep+N2FM3+N9H9
1477.608	4	pep+N2FM3+N9H9F
1532.376	4	pep+N2FM3+N10H10

E

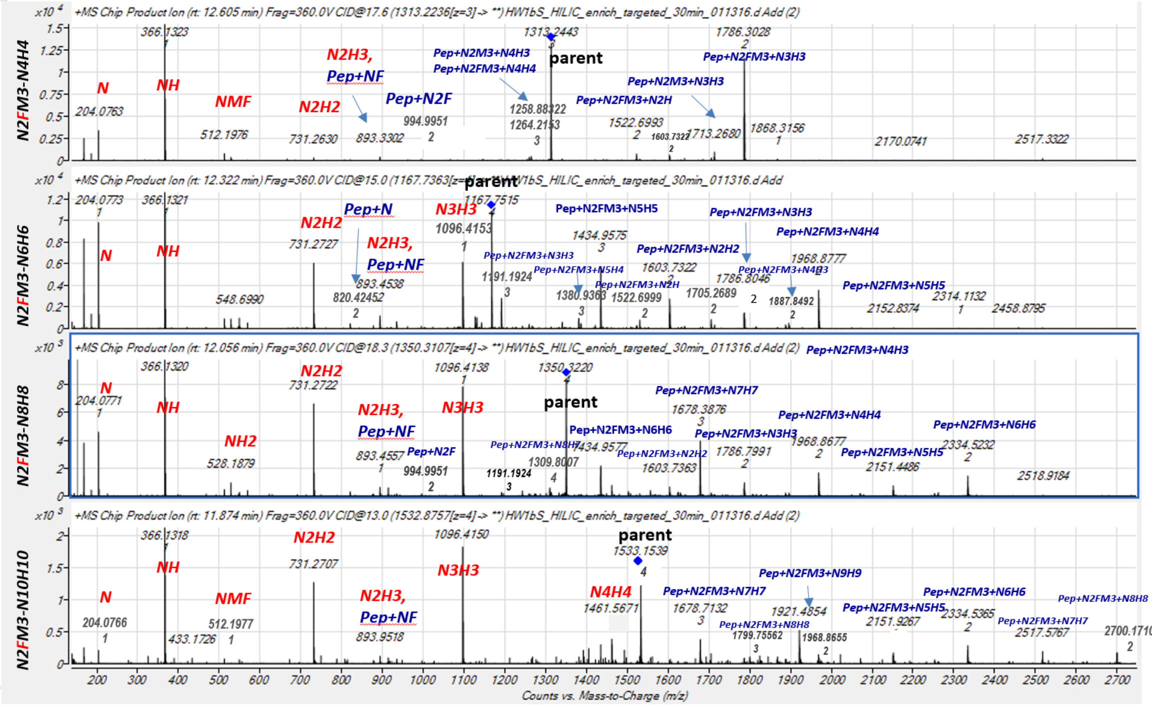

F

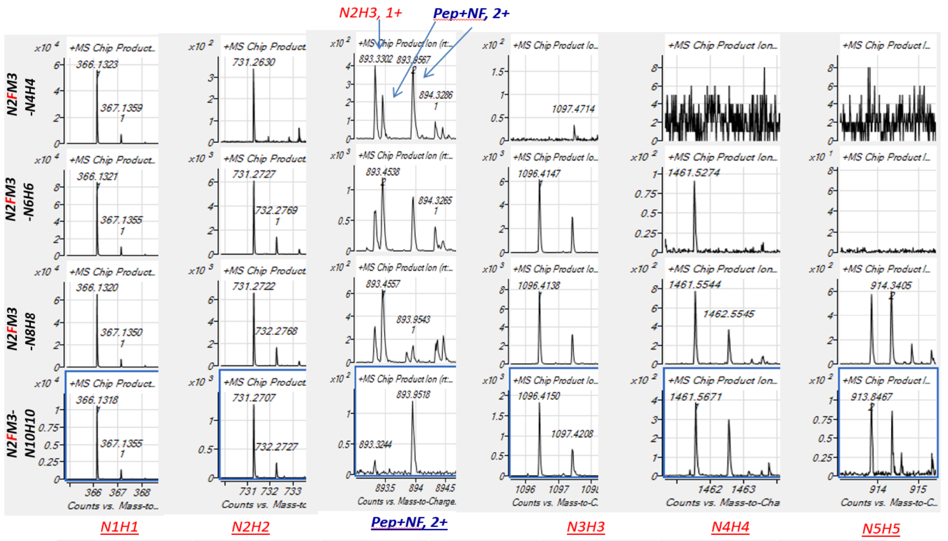

Figure S4: continued

		N1H1 (366.1392)	N2H2(731.2714)	N2H3(894.3242)	N3H3(1096.4036)	N4H4(1461.5358)	N5H5(913.8375)
N2FM3-N4H4	(a)	100.00%	0.61%	0.71%	0.00%	0.00%	0.00%
N2FM3-N6H6	(b)	100.00%	7.06%	0.76%	7.29%	0.11%	0.00%
N2FM3-N8H8	(c)	100.00%	10.15%	0.48%	12.00%	1.20%	0.92%
N2FM3-N10H10	(d)	100.00%	11.98%	0.23%	17.17%	3.58%	0.82%

Major structures >95%

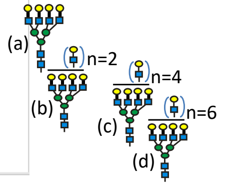

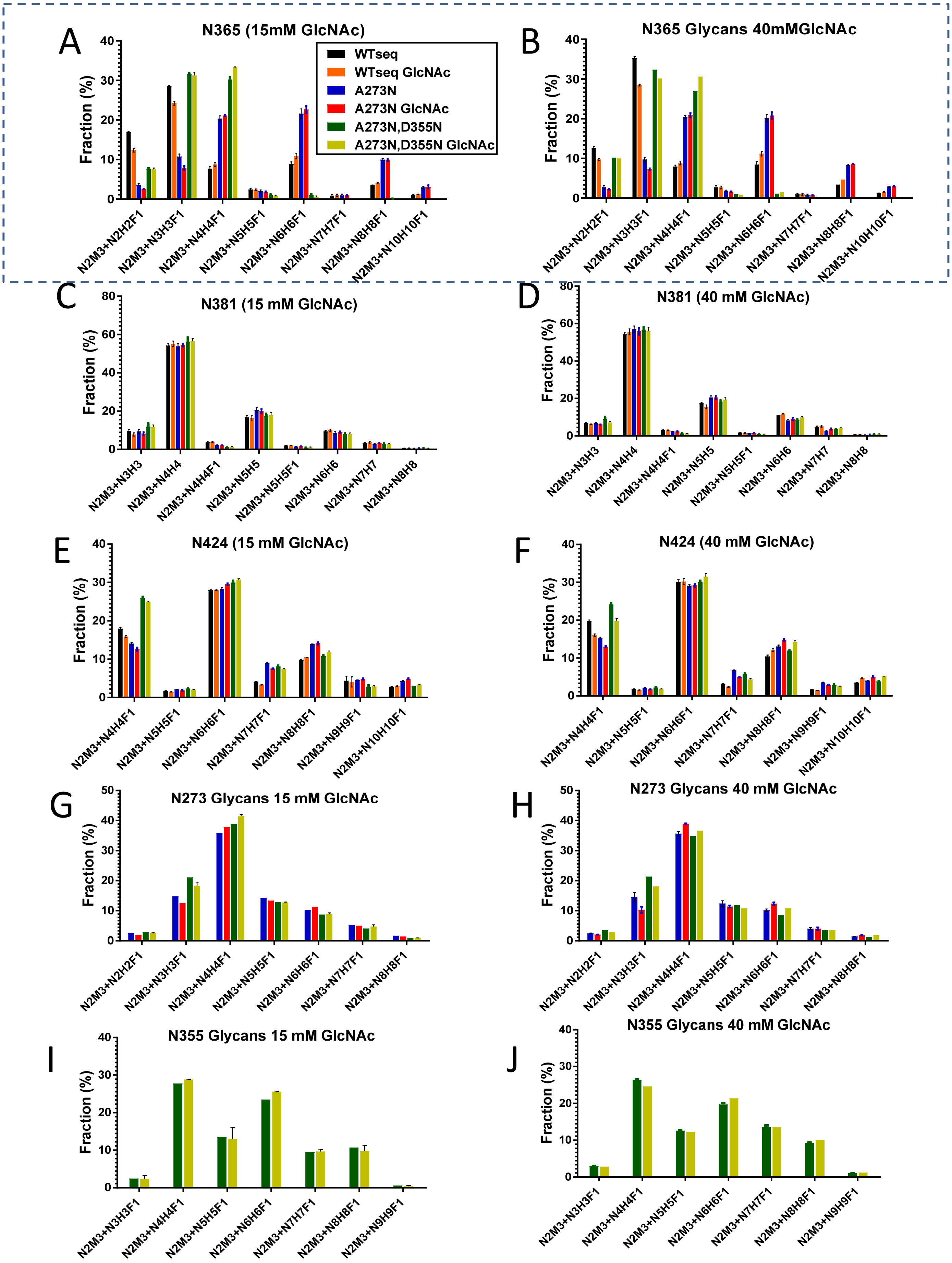

Figure S5: GlcNAc treatment increased branching selectively at N365. Dox-induced FLAG-SLC3A2 WTseq and variants cells were cultured for 48h in medium supplemented with **(A,C,E,G,I)** 15 mM GlcNAc or **(B,D,F,H,J)** 40 mM GlcNAc. GlcNAc treatment increased branching at N365 and had no effect at the other sites. Mean ± SD of 3-4 technical replicates. Added sites at A273 and D355 in human SLC3A2 has removed the N-glycans and their influence on Golgi N-glycan processing at N365.

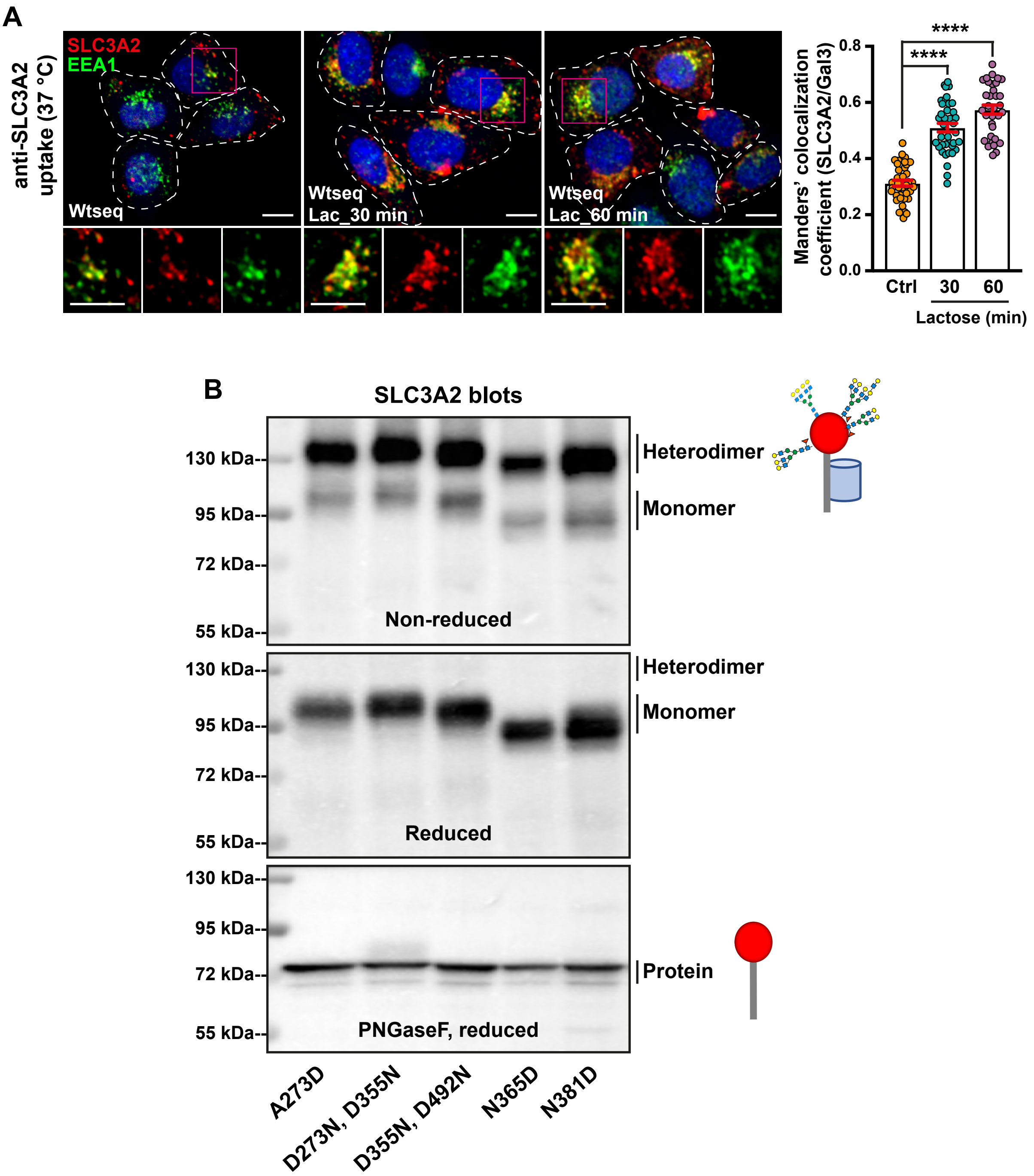

Figure S6: Prolonged galectin inhibition leads to increased SLC3A2 accumulation in perinuclear EEA1 positive endosomes. (**A**) Experiment as in Fig. 3F comparing 5 min control to prolonged lactose treatment condition, with immunolabeling of early endosomes using an anti-EEA1 antibody (green). SLC3A2 colocalization with EEA1 positive structures was quantified (histogram). Note that in the severe lactose treatment condition, the colocalization of internalized SLC3A2 with EEA1 was increased, which indicated that the protein was internalized via a different mechanism (see text for discussion). Means are ± SEM; Statistics are by one-way ANOVA, ****$p < 0.0001$. Zooms (1.8X magnification, lower panel) are from fuchsia boxed areas in the upper images. A single median plane from confocal imaging are represented. White dashed lines represent the cell contour. Nuclei in blue (DAPI). Scale bars = 10 µm. (**B**) Western blot of dox-induced FLAG-SLC3A2, non-reduced, reduced, and reduced with PNGase digestion. The majority of FLAG-SLC3A2 is disulfide-linked as heterodimers and SLC7A5 is the top partner by LC-MS/MS.

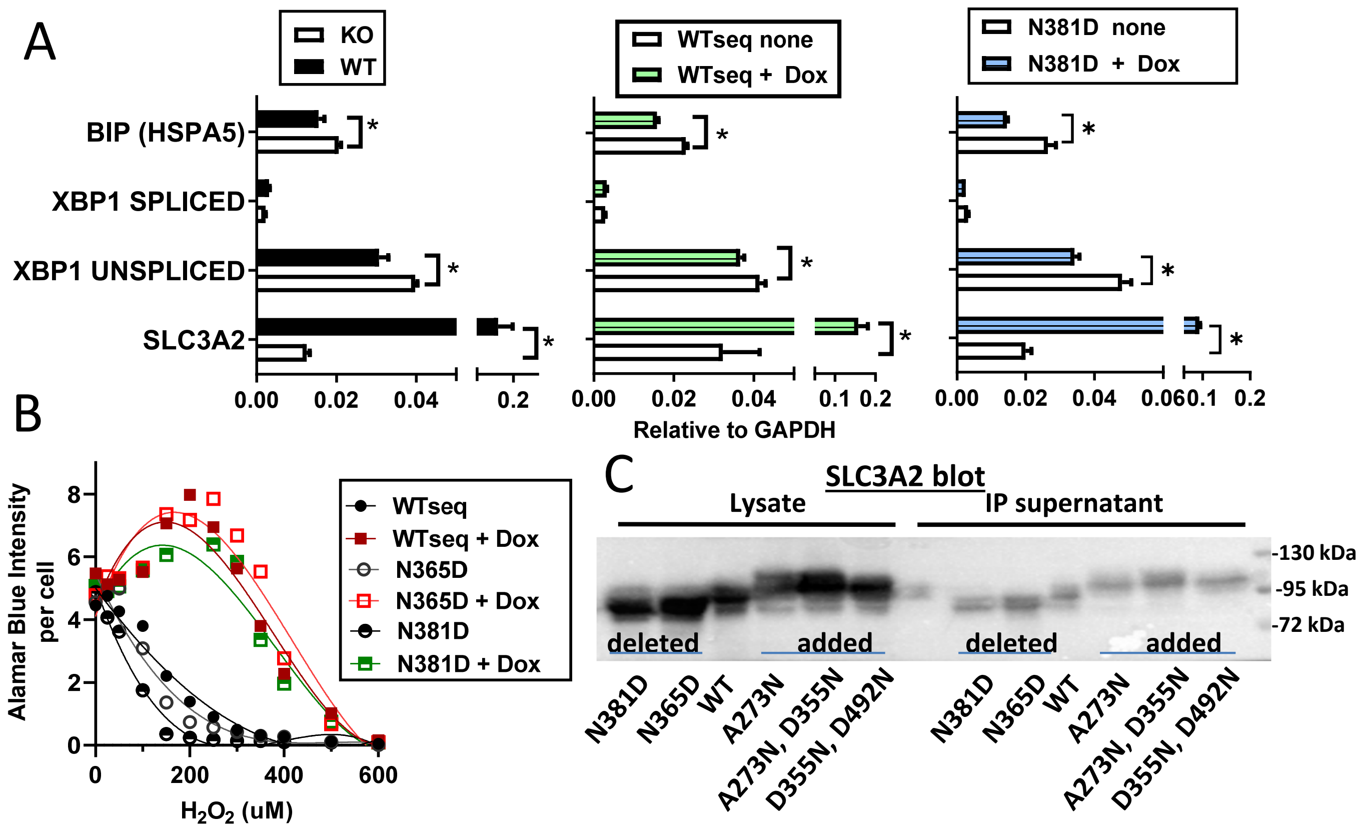

Figure S7: Dox-induced FLAG-SLC3A2 promotes resistance to ox-stress (A) Transcript levels by qPCR for stress inducible genes in WT and KO, Dox-induced WTseq and N381D. Cells were cultured in normal DMEM + 10% FCS conditions. **(B)** Sensitivity to H_2O_2. WTseq and variants +/- Dox. The Alamar Blue signal was normalized to morphologically-intact cells counted by InCell imaging. **(C)** Similar efficiency of FLAG-SLC3A2 pulldown for WTseq and site variants.

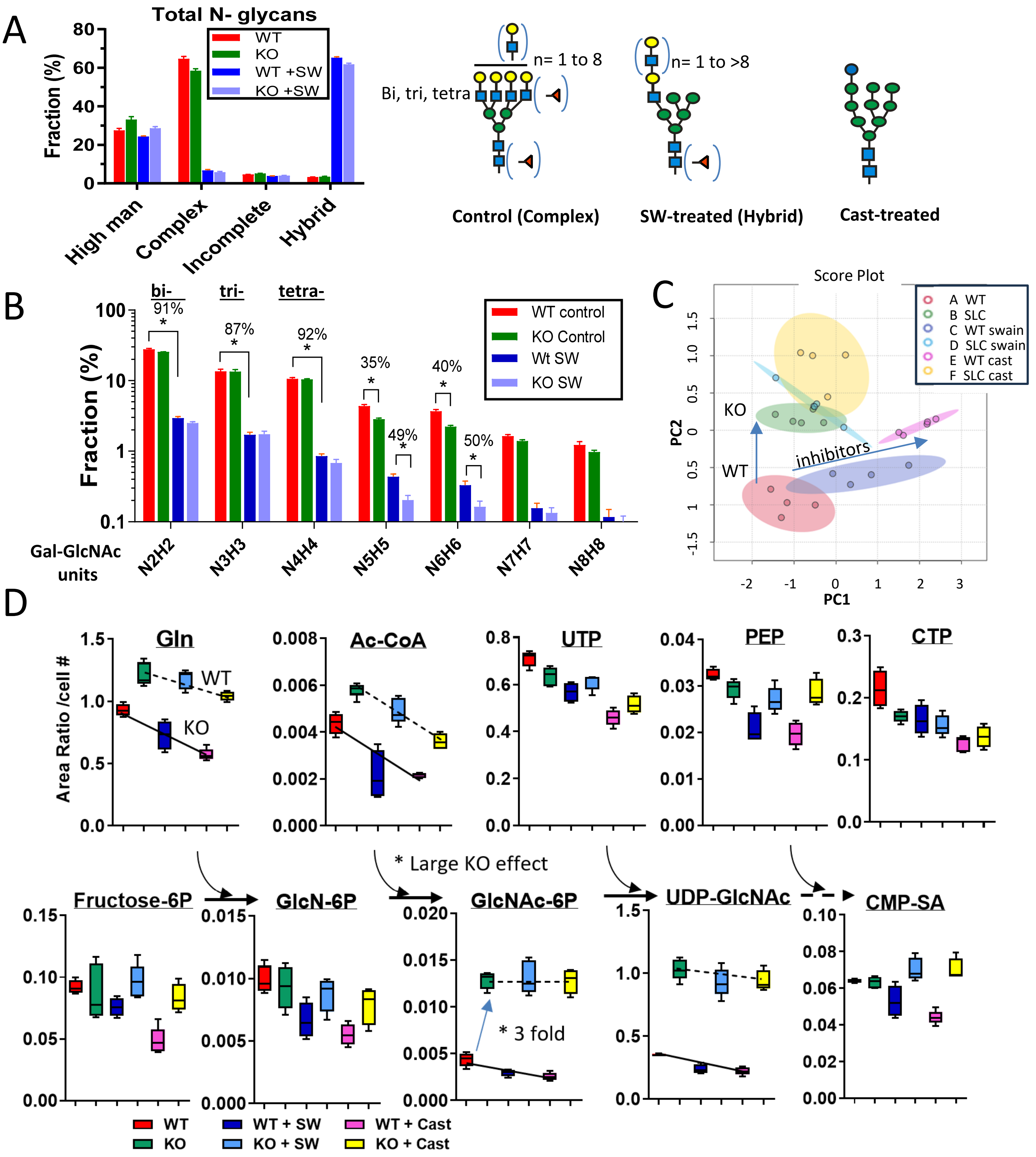

Figure S8: Golgi N-glycan processing and feedback to HBP and AA balance. SLC3A2 WT and KO HeLa were grown in DMEM + 10%FCS with and without 250 ng/ml swainsonine and 10 ug/ml castospermine for 48h, then prepared for metabolite extraction and analysis by LC-MS/MS. **(A)** **F**our classes of N-glycans Castanospermine also suppressed hybrid; (data not shown). **(B)** Branched N-glycans (bi, tri, tetra and extension with poly LacNAc (NH units). Comparisons are % decrease. Galactose (yellow circle), GlcNAc (blue square), mannose (green circle), fucose (red triangle). **(C)** Principal component analysis for 109 metabolites separates effects of SLC3A2 KO and inhibitor swainsonine and castospermine . **(D)** Effects of swainsonine and castospermine on Hexosamine biosynthesis pathway (HBP) metabolite levels.

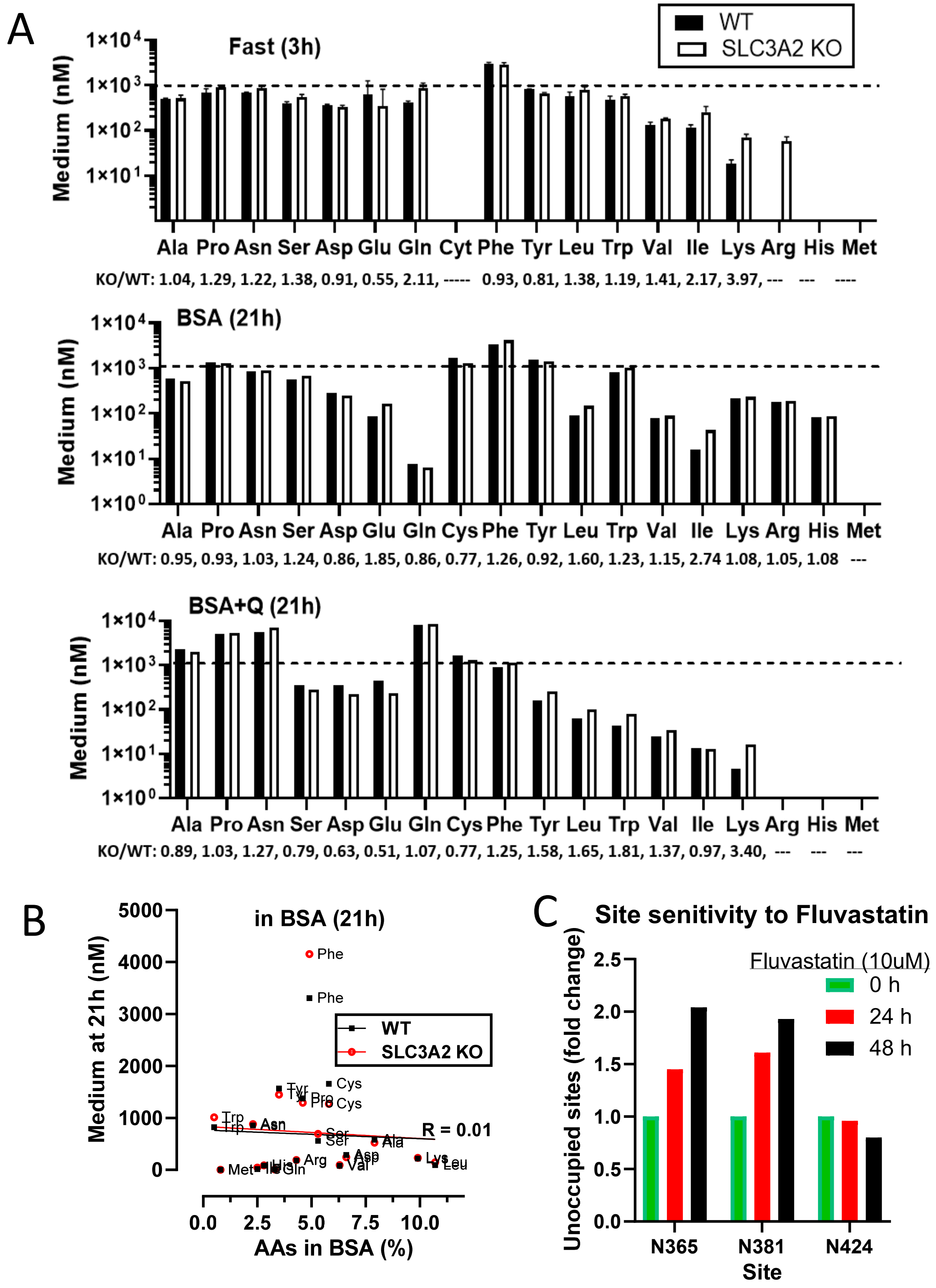

Figure S9: BSA catabolism as source of AAs. (A) Amino acid levels in WT and SLC3A2 KO cell cultured in AA-free medium for 3h, followed by addition of BSA or BSA+Gln. Standard curves for each AA were used to calculate molar amounts. **(B)** Levels **c**onsistent with cellular control over release, AA levels in medium after 21 hours did not correlate with the AA composition of BSA. **(C)** Fluvastatin-induced increases in unoccupied sites in SLC3A2 WTseq at N365, N381 and N424 by LC-MS/MS.

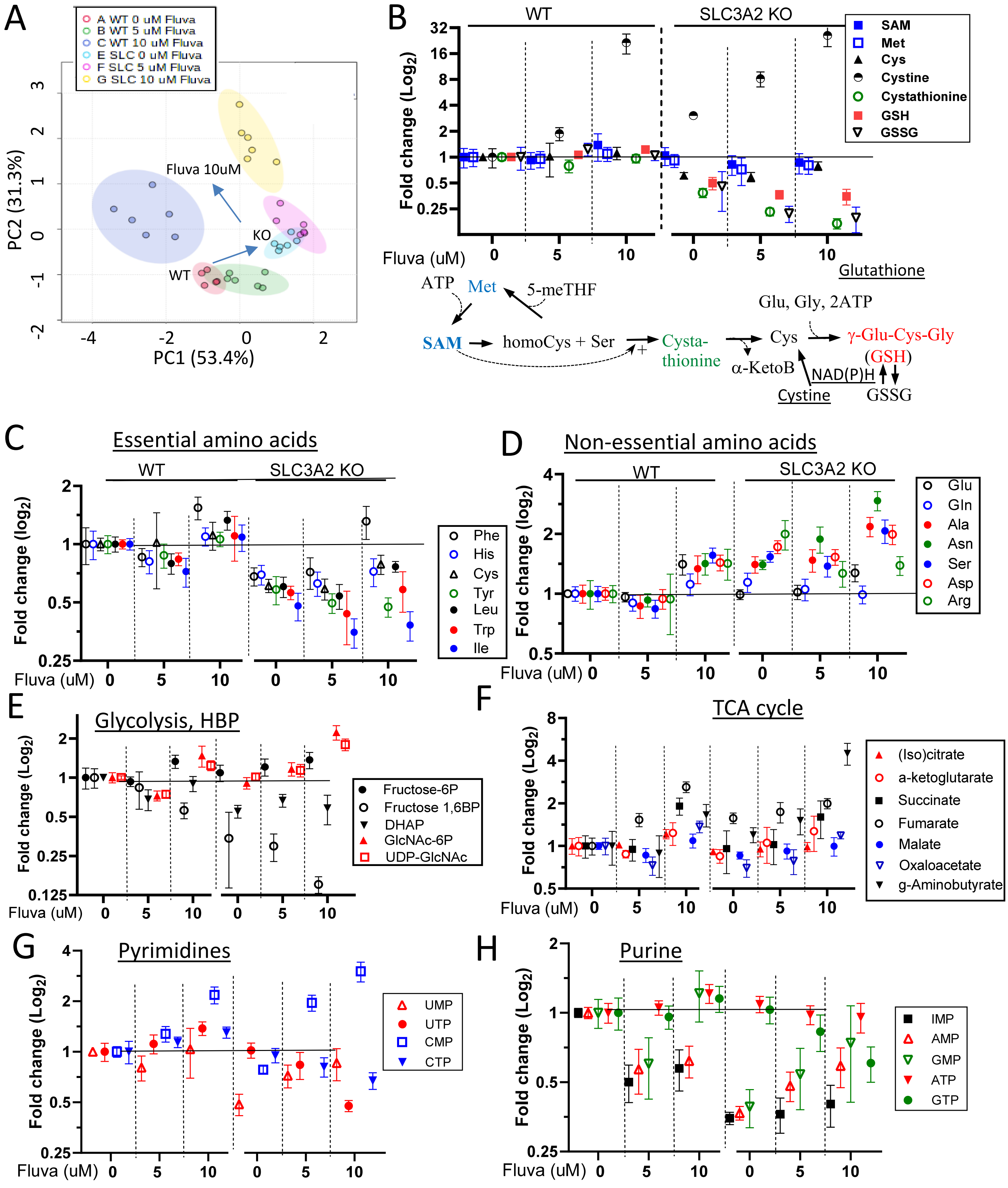

Figure S10: Metabolic profile of Fluvastatin treated WT and SLC3A2 KO cells. **(A)** Principal Component Analysis for metabolites from WT and SLC3A2 KO cells treated with 0, 5, 10 μM fluvastatin for 24h, measured by LC-MS/MS, mean ± SD (n=6). **(B)** Cystine levels were increased in SLC3A2 KO HeLa cells and further upon fluvastatin treatment, but GSH and GSSH levels were depleted, perhaps due to reduced levels of NADH which is required for conversion of Cystine to Cys. **(C-H)** Cells were treated with fluvastatin for 24h. Intracellular metabolites were measured by LC-MS/MS and displayed as KO/WT ratios, mean ± SD (n=6 technical replicates).
